# Timing-Dependent Actions of NGF Required for Cell Differentiation

**DOI:** 10.1371/journal.pone.0009011

**Published:** 2010-02-02

**Authors:** Jaehoon Chung, Hiroyuki Kubota, Yu-ichi Ozaki, Shinsuke Uda, Shinya Kuroda

**Affiliations:** Department of Biophysics and Biochemistry, Graduate School of Science, CREST, Japan Science and Technology Agency, University of Tokyo, Tokyo, Japan; University of São Paulo, Brazil

## Abstract

**Background:**

Continuous NGF stimulation induces PC12 cell differentiation. However, why continuous NGF stimulation is required for differentiation is unclear. In this study, we investigated the underlying mechanisms of the timing-dependent requirement of NGF action for cell differentiation.

**Methodology/Principal Findings:**

To address the timing-dependency of the NGF action, we performed a discontinuous stimulation assay consisting of a first transient stimulation followed by an interval and then a second sustained stimulation and quantified the neurite extension level. Consequently, we observed a timing-dependent action of NGF on cell differentiation, and discontinuous NGF stimulation similarly induced differentiation. The first stimulation did not induce neurite extension, whereas the second stimulation induced fast neurite extension; therefore, the first stimulation is likely required as a prerequisite condition. These observations indicate that the action of NGF can be divided into two processes: an initial stimulation-driven latent process and a second stimulation-driven extension process. The latent process appears to require the activities of ERK and transcription, but not PI3K, whereas the extension-process requires the activities of ERK and PI3K, but not transcription. We also found that during the first stimulation, the activity of NGF can be replaced by PACAP, but not by insulin, EGF, bFGF or forskolin; during the second stimulation, however, the activity of NGF cannot be replaced by any of these stimulants. These findings allowed us to identify potential genes specifically involved in the latent process, rather than in other processes, using a microarray.

**Conclusions/Significance:**

These results demonstrate that NGF induces the differentiation of PC12 cells via mechanically distinct processes: an ERK-driven and transcription-dependent latent process, and an ERK- and PI3K-driven and transcription-independent extension process.

## Introduction

The rat pheochromocytoma cell line (PC12) has been extensively used to study neuronal differentiation. PC12 cells respond to nerve growth factor (NGF) by differentiating into sympathetic neuron-like phenotypes characterized by neurite extension and the expression of many neuronal-specific proteins [1]–[3]. The molecular signaling pathways involved in PC12 differentiation have also been vigorously explored over the past decades. NGF binds to its high-affinity receptor, tyrosine kinase A (TrkA), followed by the phosphorylation of TrkA. Phosphorylated TrkA then transduces the signal to a variety of signaling effectors, such as phosphatidylinositol 3-kinase (PI3K) and extracellular signal-regulated kinase (ERK) [4]–[6]. Numerous reports have described the requirement for continuous NGF treatment for the successful differentiation of PC12 cells, the continuous activation of ERK for the induction of neuronal differentiation and growth arrest [2], [7]–[8], and the continuous exposure to NGF for both the survival and phenotypic maintenance of the differentiated state [9]. However, the underlying mechanism responsible for the continuous requirement of NGF stimulation remains unclear because the continuous addition of agonists and inhibitors has been performed in most studies.

Studies on the requirement for continuous stimulation in some biological processes have provided deeper insight into the mechanisms of regulation in certain cell types. For example, many studies have shown that the continuous exposure of growth factors until about 2 h before cells enter S phase is necessary for serum-arrested fibroblasts to actually enter S phase [10]. Previous studies have suggested different underlying mechanisms for this continuous stimulation requirement. One mechanism is that the continuous activation of ERK is required for the sustained down-regulation of anti-proliferative genes until the onset of S phase allows successful G1 phase progression [11]. Another is that the continuous requirement for a growth factor can be replaced with two short pulses of mitogen, one at time zero and the other at 8 h, because growth factors commit arrested cells to the cell cycle using distinct sets of signaling enzymes at two different times during the G0 to S interval. The first stimulation of growth factor is mediated by a combination of the ERK pathway and an elevation of c-Myc, whereas the second stimulation was rescued by synthetic PI3K lipid [12]. These studies suggest that the requirement for continuous or discontinuous stimulation depends on whether the continuous or discontinuous activation of downstream molecules is necessary for a particular biological process.

The de novo initiation of neurite extension after treatment with NGF is preceded by a lag in PC12 cell differentiation [3]. Therefore, this differentiation process is expected to consist of latent and extension processes. From this point of view, we hypothesized that NGF stimulation is separately required at a minimum of two discrete time points for latent and extension processes during the differentiation of PC12 cells.

Our goal in this study was to identify the underlying mechanisms explaining the requirement for continuous NGF stimulation for PC12 cell differentiation. To address this question, we performed a discontinuous stimulation assay consisting of a first (transient) stimulation followed by an interval and then a second (sustained) stimulation; we then examined whether continuous NGF stimulation could be replaced by discontinuous stimulation. We found that PC12 cells differentiated normally upon discontinuous stimulation. The first stimulation did not induce neurite extension. However, when the first stimulation was followed by the second stimulation, the combined stimulations induced a faster initial extension of neurites than when only the second stimulation was applied. The action of the first stimulation was mediated by the activities of ERK and transcription, but not PI3K activity, whereas the induction of neurite extension by the second stimulation was mediated by the activities of ERK and PI3K, but not transcriptional activity. Furthermore, we identified up- and down-regulated genes that were potentially involved in the latent process; these genes were induced by the first NGF stimulation and pituitary adenylate cyclase-activating peptide (PACAP), which could replace NGF during the first stimulation, and were mediated by ERK, but not PI3K, activity. These results demonstrated that the differentiation of PC12 cells is induced by two discontinuous stimulations that separately induce the latent process and the extension process by activating different signaling pathways.

## Materials and Methods

### Cell Culture

PC12 cells (kindly provided by Masato Nakafuku, Cincinnati Children's Hospital Medical Center, Ohio) (8×10^5^) were maintained in Dulbecco's modified Eagle's medium containing 5% horse serum and 10% bovine calf serum (Nichirei) at 5% CO_2_, 37°C. For the NGF treatment, PC12 cells were plated on poly-l-lysine-coated dishes in complete medium for 24 h and then switched to complete medium supplemented with the indicated dose of NGF (R&D), 100 nM of PACAP (Sigma), 50 ng/mL of basic fibroblast growth factor (bFGF) (Sigma), 50 ng/mL of epidermal growth factor (EGF) (Roche), 10 nM of insulin (Sigma) or 10 µM of forskolin (Sigma). The medium and the NGF were replenished every 24 h. For the pharmacological inhibition of TkrA, MEK1, PI3K and transcriptional activity in naïve cells, the cultures were pretreated with 200 nM of k252a (Sigma), 50 µM of U0126 (Promega), 50 µM of LY294002 (Sigma) and 50 µM of 5,6-dichloro-l-8-D-ribofuranosylbenzimidazole (DRB) (Sigma), respectively, for 20 min before the addition of NGF. For the control, the cells were treated with an equal volume of vehicle (dimethyl sulfoxide). To wash out stimulants or inhibitors in compounds contained in the culture medium, the culture medium was replaced with an equal volume of complete medium four times.

### Quantitative PCR Experiment

Total RNA was isolated from the PC12 cells using the RNeasy Mini Kit (Qiagen) according to the manufacturer's protocol. Complementary DNA (cDNA) was synthesized with the Power SYBR Green Cells-to-Ct kit (Ambion) according to the manufacturer's protocol. Real-time PCR was performed on cDNA in the presence of Power SYBR Green PCR Master Mix (Applied Biosystems) along with the following specific primers: ACTB (β-actin) forward primer 5′-CCCGCGAGTACAACCTTCT-3′ and reverse primer 5′-CGTCATCCATGGCGAACT-3′, neurofilament light subunit (NF-L) forward primer 5′-AGACATCAGCGCCATGCA-3′ and reverse primer 5′-TTCGTGCTTCGCAGCTCAT-3′, transforming growth factor-β (TGFβ) forward primer 5′-GAGGTGACCTGGGCACCAT-3′ and reverse primer 5′-GGCCATGAGGAGCAGGAA-3′, vaccinia growth factor (VGF) forward primer 5′-GCTCGAATGTCCGAAAACGT-3′ and reverse primer 5′-ACACTCCTTCCCCGAACTGA-3′, polo-like kinase 2 (Plk2) forward primer 5′- GCCCCACACCACCATCA-3′ and reverse primer 5′- GGTCGACTATAATCCGCGAGAT-3′, poliovirus receptor (PVR) forward primer 5′- ATGAGTGTCAGATTGCCACGTT-3′ and reverse primer 5′- TCGGGCGAACACCTTCAG-3′, plasminogen activator, urokinase receptor (Plaur) forward primer 5′- GGCTGGACCCAGGAACTTTT-3′ and reverse primer 5′- CGCCTGTCCTCAAAGATGGA-3′. The real-time PCR reactions were performed using the 7300 Real Time PCR System (Applied Biosystems). Each primer set was designed using Primer Express software (Applied Biosystems). The cDNA-generated signals for target genes were internally corrected with the ACTB cDNA signal to compensate for variations in amounts of input messenger RNA (mRNA). The gene expression level was then compared with a corresponding control sample group, and the level of regulation was determined with the 2^-ΔCt^ method according to Applied Biosystems' instructions.

### RNA Isolation and Microarray Experiments

Total RNA was extracted and purified with the RNeasy Mini Kit (Qiagen), according to the manufacturer's instructions. The concentration of isolated RNA was determined using an ND-1000 spectrophotometer (Nanodrop), according to the manual's instructions. The integrity and quantity of the isolated total RNA were assessed using an Agilent 2100 Bioanalyzer (Agilent), according to the manual's instructions. Six hundred nanograms of total RNA was converted into labeled complementary RNA (cRNA) with nucleotides coupled to a fluorescent dye using the Low RNA Input Fluorescent Linear Amplification Kit (Agilent Technologies). The quality and quantity of the resulting labeled cRNA were assessed with an ND-1000 spectrophotometer and an Agilent 2100 Bioanalyzer. Individually labeled cRNAs were not pooled before hybridization. Cy3-labeled cRNAs were hybridized to Agilent Rat Whole Genome Oligo Microarrays for 13 h at 65°C. The hybridized microarrays were then washed according to the manufacturers' recommended conditions and scanned with an Agilent G2505C scanner. Data were extracted from the scanned image with Agilent Technologies' Feature Extraction software, version 10.5.1.1. The extracted data were analyzed with GeneSpring GX 7.3.1 software (Silicon Genetics). Agilent standard scenario normalizations for FE1-color arrays were applied to all data sets. A subset of genes for data interrogation was generated; spots of poor quality and gene probes that were present in 9 of the 18 arrays (duplicates of continuous NGF, transient PACAP, transient insulin and transient NGF with U0126 or LY294002 stimulations, and tetracates of control and transient NGF stimulations) were excluded. The relative expression levels of each probe for all the stimulation sets, compared with the control, were determined based on the average expression value of each probe; probes differentially expressed by greater than 4.0-fold were selected. A GO enrichment analysis was performed with the Web-based GOEAST software toolkit and a significance threshold of *p* = 0.05 [13]. The microarray data was submitted to the Gene Expression Omnibus (GEO; http://www.ncbi.nlm.nih.gov/geo) with the series accession number GSE18016.

### Quantitative Analysis of Neurite Extension

PC12 cells were fixed with 3.7% paraformaldehyde (Wako) in phosphate-buffered saline (PBS) for 15 min. Cultures were washed with PBS, incubated with 1 µg/mL of Hoechst solution (Invitrogen) and 1 µg/mL of CellMask (Invitrogen) in PBS for 1 h at room temperature, and then washed with PBS. The resulting images were captured on a CellWoRx (Thermo Scientific). Using the CellMask signal as the neuronal image and the Hoechst signal as the a nuclear image, we measured the length of the neurites using the NeuroTracer, Image J plug-in [14] (**see Figure S7**). The length of each neurite was expressed as the average neurite length (in microns) of a single cell. We used the length of the neurites to indicate the neurite extension level, instead of counting the number of cells whose neurite length exceeded a certain level, since neurite length is more quantitative than cell numbers counted with an arbitrary threshold criterion.

### Immunostaining

PC12 cells were fixed with 3.7% paraformaldehyde (Wako) in PBS, permeabilized with 0.1% Triton X-100 in PBS**,** and blocked with 5% bovine serum albumin in PBS for 30 min at room temperature. An antibody directed against phospho-ERK (Cell Signaling), phospho-Akt (Cell Signaling) or phospho-cAMP response element binding (CREB) (Cell Signaling) was added to CanGetSignal (Toyobo) for 1 h at room temperature. The cultures were washed with PBS; incubated with an Alexa Fluor 488-conjugated goat anti-mouse antibody (Invitrogen), Alexa Fluor 488-conjugated goat anti-rabbit antibody (Invitrogen), Hoechst solution, and CellMask in PBS for 1 h at room temperature; and then washed with PBS. The resulting images were captured on a CellWoRx (Thermo Scientific). The captured images were analyzed with vHCS (Thermo Scientific).

### Immunoblotting

Cell lysates were subjected to standard SDS-PAGE (acrylamide:bis = 29.5∶1) and then transferred to a nitrocellulose membrane. The membranes were probed with anti-phospho TrkA (Y490) antibody (1∶1,000; Cell Signaling Technology) or anti-ERK1/2 antibody (1∶1,000; Cell Signaling Technology). Horseradish peroxidase (HRP)-conjugated secondary antibodies (Amersham Biosciences, Piscataway, NJ) were used at a dilution of 1∶2,500, and an enhanced chemiluminescence (ECL) detection kit (Amersham Biosciences) was used for the HRP detection.

## Results

### Continuous NGF Stimulation for Induction of PC12 Cell Differentiation Can Be Divided into Two Discontinuous Stimulations

**Figure 1:**
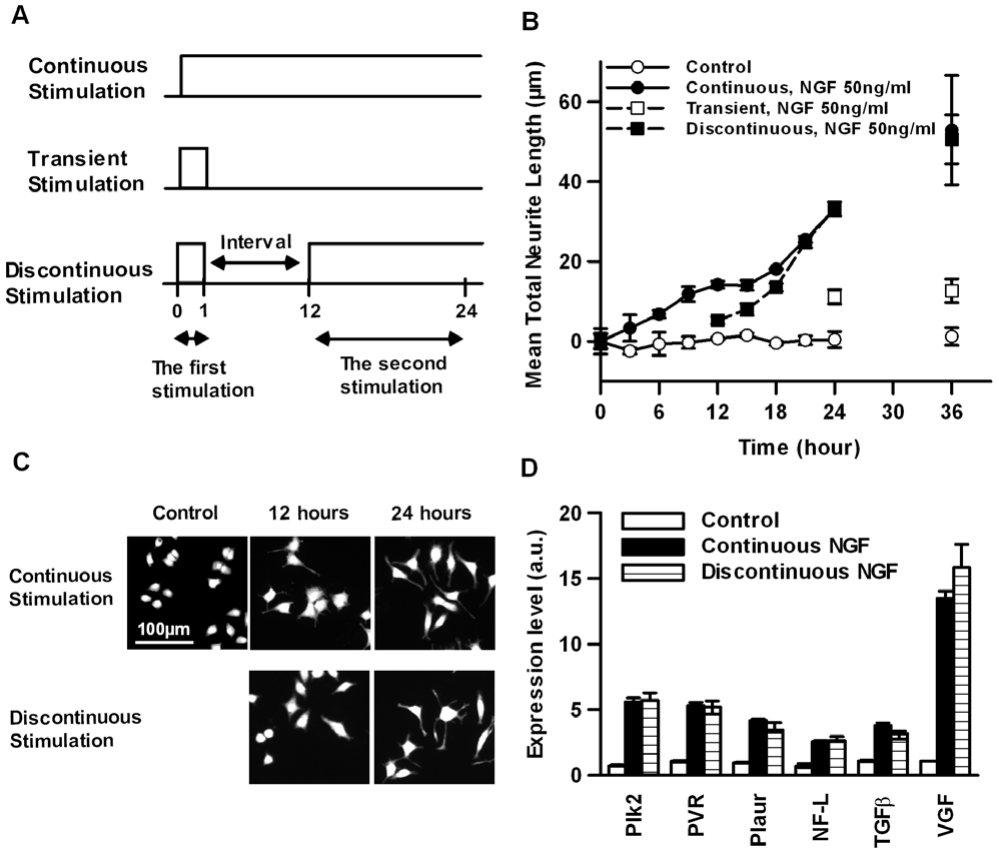
Discontinuous NGF-stimulation-induced differentiation of PC12 cells. (A) Diagram of the stimulation assays; continuous (upper), transient (middle) and discontinuous (low) stimulation. As the transient stimulation, PC12 cells were exposed to 50 ng/ml of NGF for 1 h, then NGF containing medium was replaced with NGF-free medium 4 times. The default conditions of the discontinuous NGF stimulation were as follows: the first transient stimulation (50 ng/mL NGF for 1 h) followed by an 11-h interval, and the second sustained stimulation (50 ng/mL NGF for 12 h), unless stated otherwise. (B) Length of neurite after continuous, transient or discontinuous NGF stimulation. PC12 cells were exposed to NGF-free medium (open circle) or continuous stimulation with 50 ng/mL NGF (closed circle), discontinuous stimulation with 50 ng/mL NGF (closed square) or transient stimulation with 50 ng/mL NGF for 1 h (open square). At the indicated time points, the neurite lengths were measured as described in Materials and Methods. The values represent the means ± S.E.M. (n = 3). (C) Representative images of PC12 cells after continuous or discontinuous NGF stimulation at the indicated time points. PC12 cells before stimulation were used as a control, as indicated. (D) Quantification of the mRNA expression level of differentiation marker genes. Twenty-four hours after continuous or discontinuous stimulation, the mRNA expression levels of neurofilament light subunit (NF-L), transforming growth factor-β (TGFβ), vaccinia growth factor (VGF), poliovirus receptor (PVR), polo-like kinase 2 (Plk2) and plasminogen activator, urokinase receptor (Plaur) were measured using real-time PCR. The data were normalized using the expression level of β-actin (ACTB) as an internal control. The mRNA expression levels of non-stimulated cells were used as a control. The values represent the mean fold expression compared with the control ± S.E.M. (n = 3). *p>0.05; Student *t*-test comparing continuously and discontinuously stimulated cells.

Because the de novo induction of neurite extension of PC12 cells upon NGF stimulation was preceded by a lag phase, we speculated that the differentiation process of PC12 cells might consist of two distinct processes: latent and extension. From this perspective, we hypothesized that NGF stimulation is required for both processes. To test this hypothesis, we used discontinuous NGF stimulation **(** **Figure 1A** **)** and examined cell differentiation by quantifying the extension of neurite length and changes in gene expression levels, since the differentiation of PC12 cell is characterized by neurite extension and the expression of neuronal genes [6].

We confirmed neurite extension upon continuous NGF stimulation. PC12 cells did not show a clear extension of neurite length at 12 h after NGF stimulation, but they exhibited considerable extension of neurites at 24 h **(** **Figure 1B and C** **)**. In contrast, transient NGF stimulation (1 h of 50 ng/ml NGF stimulation followed by replacing NGF-containing medium with NGF-free medium 4 times) did not induce neurite extension, indicating that transient NGF stimulation is insufficient for neurite extension. However, after stimulation with NGF again at 12 h after the onset of the transient stimulation, the neurites began to quickly extend and their length became similar to that of neurites exposed to continuous stimulation for 24 h. These results indicate that re-stimulation with NGF induced a fast initial extension of neurites. The lengths of the neurites at the 36 h time point were also similar between re-stimulated and continuously stimulated cells, and this similarity persisted for 8 days, suggesting the occurrence of the same differentiation process, at least for the observed period **(see Figure S1A).** Note that the NGF was effectively removed after the first stimulation by repetitively washing the cells with NGF-free culture media, since only a marginal level of neurite extension was observed after the first stimulation alone **(** **Figure 1B** **)**.

To confirm from a molecular chemical aspect whether the differentiation induced by discontinuous stimulation is similar to that induced by continuous stimulation, we quantified the expression levels of differentiation marker genes [15]–[20]. A quantitative polymerase chain reaction (qPCR) experiment revealed that both continuous stimulation and discontinuous stimulation induced similar levels of up-regulation for several marker genes, such as transforming growth factor-β (TGFβ), neurofilament light subunit (NF-L), vaccinia growth factor (VGF), poliovirus receptor (PVR), polo-like kinase 2 (Plk2) and the plasminogen activator urokinase receptor (Plaur) at the 24 hr time point **(** **Figure 1D** **)**. The expression levels of these genes remained similar for at least 8 days **(see Figure S1B).**

Taken together, these results suggest that the NGF-driven differentiation process of PC12 cells can be divided into two discrete processes: latent and extension. The latent process is the preparation process of neurite extension induced by the first stimulation, and the extension process is the neurite extension process induced by the second stimulation.

### Optimal Conditions for Discontinuous Stimulation

**Figure 2:**
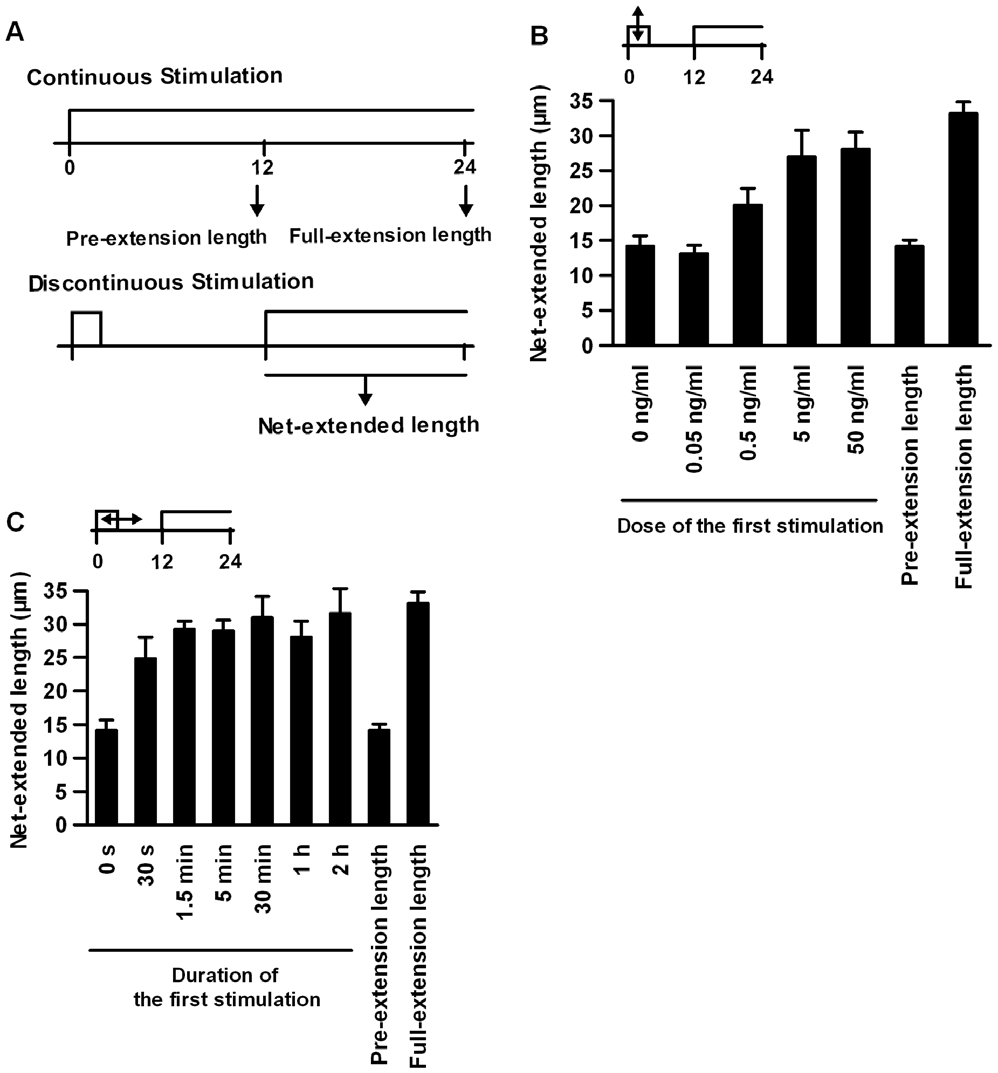
Dose- and duration-dependency of the first stimulation. (A) Evaluation of the net-extended length of the neurites. The net-extended length was defined as the length of the extended neurites during the 12-h period of the second stimulation. Also, the pre-extension length and the full-extension length were defined as the length of the neurites at 12 h and 24 h after continuous stimulation with 50 ng/mL of NGF, respectively. (B) Dose-dependency of the first stimulation. The indicated doses of NGF treated for 1 h were used as the first stimulation, and the net-extended lengths of the neurites were measured. (C) Duration-dependency of the first stimulation. The indicated durations of treatment with 50 ng/mL of NGF were used as the first stimulation, and the net-extended lengths of the neurites were measured. In both (B) and (C), a second NGF stimulation (50 ng/mL) for an additional 12 h was applied beginning at the 12-h time point. The values represent the means ± S.E.M. (n = 4).

To determine the optimal conditions for discontinuous stimulation, we examined the durations and dose dependencies of the first and second stimulations. We also determined the optimal duration of the interval between the stimulations. To evaluate the level of neurite extension for various patterns of stimulation, we measured the net-extended length of neurites during the second stimulation (hereafter, this length is denoted as “net-extended length”) and compared the net-extended lengths with the length of the neurites after 12 h of continuous stimulation (hereafter denoted as “pre-extension length”) or after 24 h of continuous stimulation (hereafter denoted as “full-extension length”) **(** **Figure 2A** **)**.

To determine the appropriate conditions for the first transient stimulation, we examined the dose- and duration-dependency of the first stimulation. The first transient stimulation was required so that the neurites would undergo immediate and fast extension in response to the second stimulation. We examined the dose-dependency of the first stimulation **(** **Figure 2B** **)**. The net-extended length during the first NGF stimulation at a dose of 0.05 ng/mL was similar to the pre-extension length. As the NGF dose increased during the first stimulation, the net-extended lengths increased and reached a maximum length at doses higher than 5 ng/mL. The saturation level of the net-extended length was similar to that of the full-extension length, indicating that a concentration of NGF higher than 5 ng/mL was sufficient to produce a maximal effect. Furthermore, we measured the distribution pattern of neurite length upon continuous or discontinuous stimulation in a dose-dependent manner **(see Figure S2B).** The distribution patterns before and 12 h after the transient first stimulation were similar **(see Figure S2A,D)**. Upon the second stimulation, the neurites were extended, resulting in a decrease in the number of short-neurite-bearing cells and an increase in the number of long-neurite-bearing cells with neurite lengths of longer than about 20 µm **(Figure S2 D,E)**. However, when a suboptimal dose (0.5 ng/mL) was used for the first stimulation, the population of long-neurite-bearing cells with neurite lengths of longer than about 40 µm was smaller than that upon stimulation under optimal conditions when counted at the 24-h time point, indicating that the dose of the first stimulation affected the potency of neurite extension **(Figure S2E,F,H).** We also examined the duration-dependency of the first stimulation **(** **Figure 2C** **)**. As the duration of the first stimulation increased, the net-extended length increased and reached a maximal effect at a duration of longer than 1.5 min. The maximal net-extended length was similar to the full-extension length. This result indicates that 1.5 min is a sufficient duration for the first stimulation. Taken together, these results indicate that a first NGF stimulation at a dose higher than 5 ng/mL and a duration longer than 1.5 min is sufficient to produce a maximal effect.

**Figure 3:**
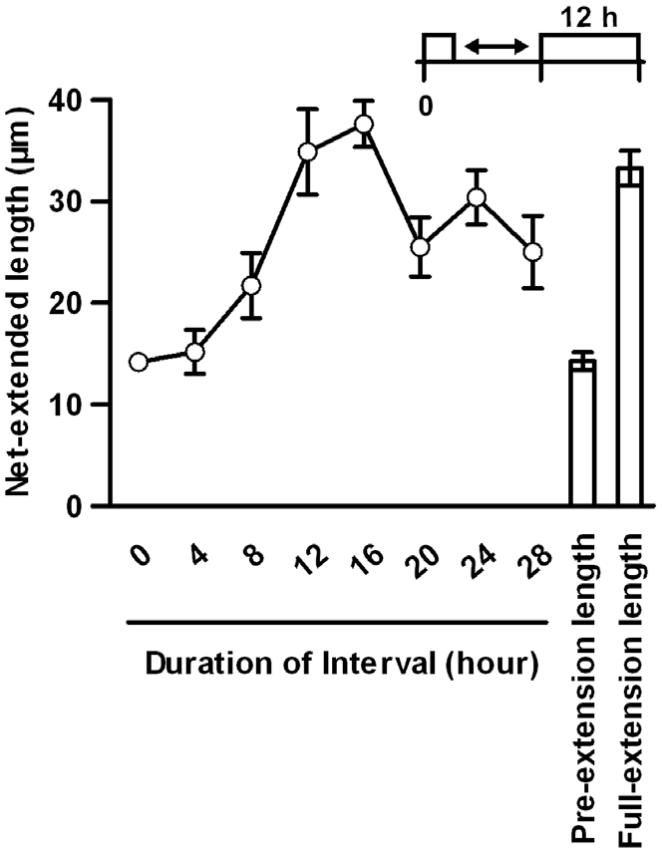
Duration-dependency of the interval. The indicated interval durations were used and the net-extended lengths were measured. The first stimulation with NGF (50 ng/mL NGF for 1 h) (open circle) and the second stimulation with NGF (50 ng/mL NGF for 12 h) were performed as before. The values represent the mean ± S.E.M. (n = 4).

The interval after the first stimulation consists of a period during which particular processes required for the acquisition of a fast response to the second stimulation occur. To examine how the duration of this interval affects further neurite extension upon the second stimulation, we altered the durations of the interval **(** **Figure 3** **)**. As the duration increased from 0 to 12 h, the net-extended length gradually increased. This effect reached a maximal effect at around 12 to 16 h after the first stimulation and then decreased with longer interval durations. This result indicates that a 12- to 16-h interval represents the optimal duration and also suggests that the action of the first stimulation is reversible.

**Figure 4:**
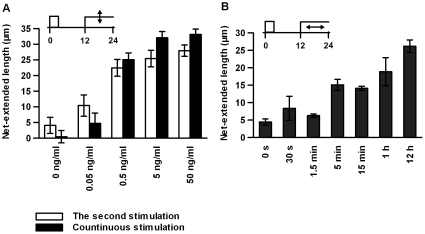
Dose-and duration-dependency of the second stimulation. (A) The indicated doses of NGF were given for 12 h beginning at the 12-h time point. Continuous stimulations with the indicated doses of NGF for 24 h are also indicated. (B) The indicated durations of the treatment of 50 ng/mL of NGF are shown from the 12-h time point. A dose of 50 ng/mL of NGF for 1 h was used as the first stimulation, and the net-extended lengths between the 12- and 24-h time points were measured (A, B). The values represent the mean ± S.E.M. (n = 3).

To determine the appropriate conditions for the second stimulation, we changed the doses and durations of the second stimulation **(** **Figure 4** **)**. As the dose of the second stimulation increased, the net-extended length increased and reached a maximal effect at a dose higher than 0.5 ng/mL **(** **Figure 4A** **)**. Of note, the dose-dependency of the second stimulation was very similar to that of the continuous stimulation. We also changed the duration of the second stimulation and examined the net-extended length at 12 h after the onset of the second stimulation **(** **Figure 4B** **)**. The net-extended length increased, along with the increase in duration, and reached a maximum length after 12 h of stimulation. One hour of the second stimulation was insufficient to induce full-length neurite extension. The neurite extension rate upon the transient second stimulation was slower than that upon the sustained second stimulation, although gradual neurite extension was observed after the removal of NGF for up to at least 12 h **(see Figure S2J)**. Concerning the distribution pattern of the neurite lengths, the population of long-neurite-bearing cells was much smaller than the population under optimal conditions, whereas the population of short-neurite-bearing cells decreased upon a transient (1 h) second stimulation, indicating that sustained stimulation was required for the prolonged extension of the neurite**s** **(see Figure S2D,G,I).**

These results revealed that the differentiation process of PC12 cells consists of a latent process, which is induced by transient stimulation followed by a 12-h interval, and an extension process, which requires additional sustained stimulation after the first stimulation. Hereafter, we used the following stimulation patterns for discontinuous stimulation: 50 ng/mL of NGF for 1 h as the first stimulation (representing a sufficient dose and duration to induce the latent process) followed by an 11-h interval and a second stimulation of 50 ng/mL of NGF for 12 h (representing a sufficient dose and duration to induce the extension process and to allow the effects of the latent process to be evaluated), unless specified otherwise.

### Signaling Pathways Required for the First and Second Stimulation

To explore the downstream signaling pathways underlying the actions of the first and second stimulations, we examined the effects of pharmacological inhibitors on the actions of the first and second stimulations. Because ERK and PI3K are well-known signaling molecules involved in the NGF-dependent differentiation of PC12 cells [6], [21], we examined the effect of U0126 or LY294002, specific inhibitors of ERK and PI3K activity, respectively, on the actions of the first and second stimulations. We also tested the effect of k252a, an inhibitor of TrkA, on the actions of both stimulations. This approach provided novel information about the temporal requirements of ERK and PI3K for cell differentiation.

We confirmed that U0126, LY294002 and k252a inhibited the neurite extension induced by continuous NGF stimulation and the phosphorylation of ERK, PI3K and TrkA, as previously reported **(see Figure S3)** [22]–[25]. We also confirmed that the effects of the inhibitors could be reversibly removed **(see Figure S4),** indicating that the addition of inhibitors for the first transient stimulation does not directly affect the action of the second stimulation.

**Figure 5:**
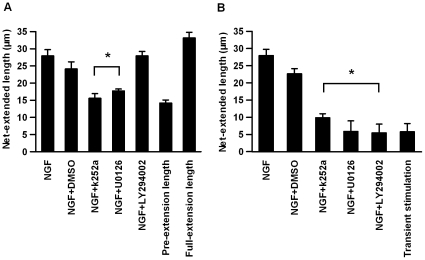
Signaling pathways required for the actions of the first and second stimulations. (A) Effect of inhibitors on the action of the first stimulation. The indicated inhibitors, k252a (200 nM), U0126 (50 nM), LY294002 (50 nM) and vehicle control (DMSO)**,** were treated beginning 20 min before the first stimulation and continuing until the 2-h time point; then, the net-extended lengths were measured. (B) Effect of inhibitors on the action of the second stimulation. The indicated inhibitors, k252a (200 nM), U0126 (50 nM), LY294002 (50 nM) and vehicle control (DMSO), were treated beginning 20 min before the second stimulation and continuing until the 24-h time point; the net-extended lengths were then measured. The lengths of the neurites at 24 h after transient (1 h) stimulation with 50 ng/mL of NGF were measured. The pre-extension lengths and the full-extension lengths are also indicated (A, B). The values represent the mean ± S.E.M. (n = 3). *p<0.05; Student *t*-test comparing DMSO- and inhibitor-treated cells. The treatment of 50 ng/mL of NGF for 1 h and 12 h were used as the first and second stimulations, respectively.

We added the indicated inhibitors during the first stimulation and quantified the net-extended lengths **(** **Figure 5A** **)**. The net-extended lengths with the addition of U0126 or k252a were shorter than those of neurites not exposed to the inhibitors and were similar to the pre-extension lengths. This result indicates that U0126 or k252a almost completely inhibited the action of the first stimulation. In contrast, the net extended-lengths with the addition of LY294002 were similar to the full-extension lengths. This result indicates that LY294002 does not affect the action of the first stimulation, because we also confirmed that ERK activation was not affected by LY294002 treatment (data not shown). These results indicate that the action of the first stimulation requires the activities of TrkA and ERK, but not PI3K activity.

We also examined the effect of the inhibitors on the action of the second stimulation **(** **Figure 5B** **)**. The net-extended lengths with the addition of U0126, LY294002 or k252a were similar to those of neurites that did not undergo the second stimulation, indicating that the action of the second stimulation requires TrkA, ERK and PI3K activities. Taken together, these results indicate that the NGF-dependent latent process is mediated by the ERK pathway but not by the PI3K pathway, whereas the extension process is mediated by the ERK and the PI3K pathways. This is the first description of the temporal requirement of signaling molecules during the cell differentiation process.

### Replacement of the First or Second Stimulants by Other Stimulants

In addition to NGF, many other stimulants have been reported to stimulate the activation of ERK, PI3K and other signaling cascades [6], [18], [26]. To gain deeper insight into the signaling mechanisms of the actions involved in the first and second stimulations, we examined whether other stimulants, such as EGF, insulin, PACAP, bFGF or forskolin, could replace NGF as the first or second stimulant. Continuous stimulation with PACAP, bFGF or forskolin was insufficient to achieve full-length extension, although these stimulants have been reported to induce differentiation in PC12 cells **(see Figure S5)** [18], [27]. Under our experimental conditions, NGF, but not the other stimulants that were tested, induced the sustained activation of downstream molecules such as ERK, Akt and CREB **(see Figure S6).** This observation might explain why stimulants other than NGF failed to induce neurite extension in the PC12 cells.

**Figure 6:**
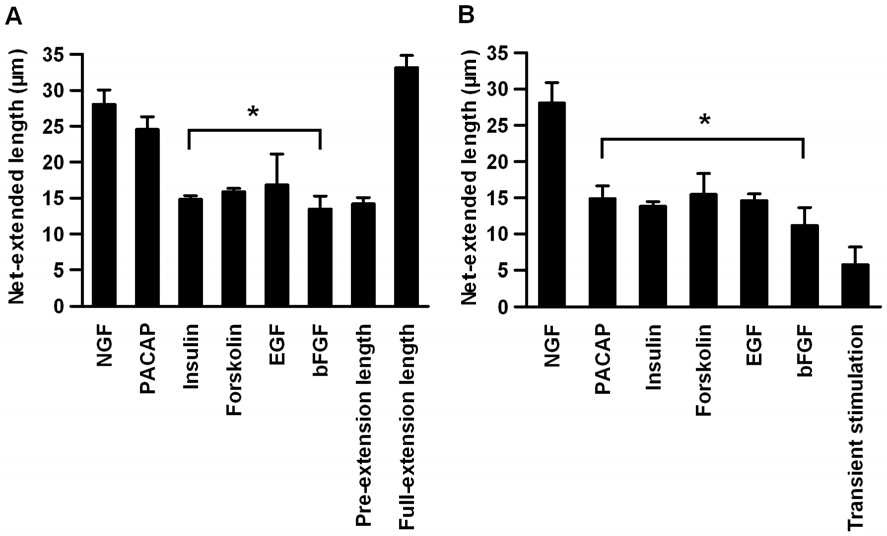
Replacement of the first or second stimulation with various stimulants. (A) The indicated stimulants were treated for 1 h as the first stimulation, and 50 ng/mL of NGF treated for 12 h was used as the second stimulation. (B) The indicated stimulants were treated for 12 h as the second stimulation, and 50 ng/mL of NGF treated for 1 h was used as the first stimulation. PACAP (100 nM), insulin (10 nM), EGF (50 ng/ml), forskolin (10 µM) and bFGF (50 ng/ml) were used in both (A) and (B). The values represent the mean ± S.E.M. (n = 3). *p<0.05; Student *t*-test comparing cells treated with NGF and the indicated stimulants.

We tested whether the other stimulants could replace NGF as the first stimulant **(** **Figure 6A** **)**. The net-extended length induced by PACAP was almost the same as that induced by NGF and almost the same as the full-extension length. However, the net-extended lengths induced by the other stimulants were almost the same as the pre-extension length. This result indicates that PACAP, but not the other stimulants, was capable of acting as the first stimulant. We also tested whether these stimulants can replace NGF as the second stimulant **(** **Figure 6B** **)**. The net-extended lengths for the other stimulants were much shorter than those induced by NGF and were slightly longer than the net-extended length achieved without the second stimulation. The effect of these stimulants might be the induction of neurite extension through marginal activation of the necessary signaling pathways related to neurite extension. Alternatively, these short extensions upon exposure to the stimulants might represent some form of morphological extrusion other than neurites, such as filopodia. This result indicates that none of the tested stimulants was fully sufficient to replace NGF as the second stimulant.

Taken together, these results indicate that PACAP can serve as the first stimulant, but possibly not as the second stimulant, indicating that different mechanisms underlie the actions of the first and second stimulations. Common features of the signaling activities of NGF and PACAP, but not of other stimulants, are sustained ERK and CREB activation, suggesting that the sustained, rather than transient, activation of ERK and CREB is critically important for the action of the first stimulation **(see Figure S6).** Additionally, PACAP did not stimulate the phosphorylation of Akt during the first stimulation **(see Figure S6C,E).** This result is consistent with the PI3K-independent action of the first stimulation **(** **Figure 5A and B** **)**.

### Requirement of Transcriptional Activity for the Action of the First Stimulation, but Not for That of the Second Stimulation

**Figure 7:**
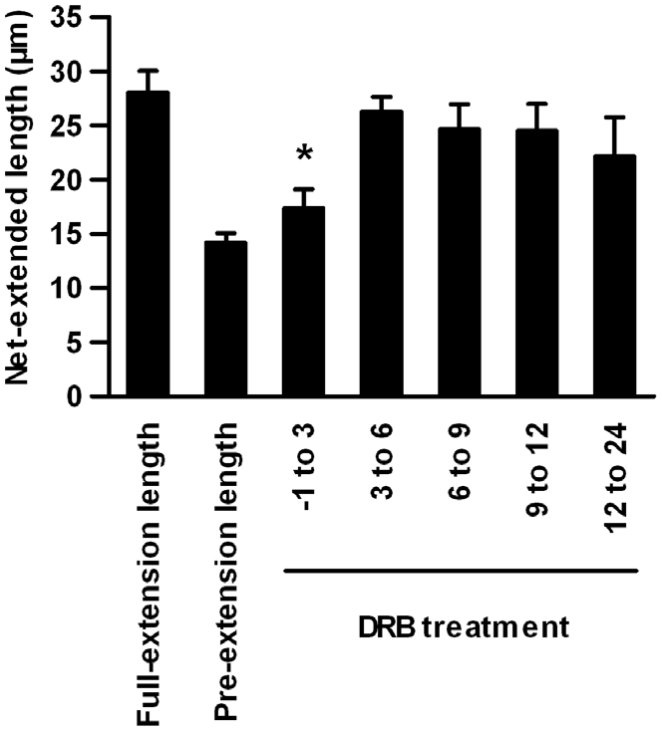
Requirement of transcriptional activity for the action of the first stimulation. The transcriptional inhibitor DRB (200 nM) was treated during the indicated time period and the net-extended lengths were measured. The values represent the mean ± S.E.M. (n = 3). *p<0.05; Student *t*-test comparing DRB-treated and untreated cells.

The results in Figure 5 and 6 indicate that ERK activity is essential for the action of the first and second stimulations. According to previous studies, transcriptional regulation is critical for the ERK-mediated differentiation of PC12 cells [28]. The action of the first stimulation is most likely mediated by transcriptional regulation. We investigated whether transcriptional activity is required for the action of the first or second stimulation by adding 5,6-dichloro-l-8-D-ribofuranosylbenzimidazole (DRB), a reversible transcription inhibitor **(** **Figure 7** **)**.

With the addition of DRB, the net-extended length during the initial 3 h was similar to the pre-extension length, indicating that the action of the first stimulation was almost completely inhibited by DRB. On the other hand, with the addition of DRB at later time points, the net-extended lengths were almost similar to the full-extension lengths, indicating that the actions of the first or second stimulation were not affected by DRB during this period. This result indicates that transcriptional activity during the initial 3 h is critically important for the action of the first stimulation, but not needed for the action of the second stimulation.

### Identification of Genes Potentially Involved in the Action of the First Stimulation

Transcriptional activity appeared to be essential for the action of the first stimulation **(** **Figure 7** **)**; therefore, we examined which genes might be required for the action of the first stimulation. To identify genes specifically involved in the action of the first stimulation, but not other differentiation processes, we used diverse patterns of stimulations to narrow down the candidate genes.

Genes regulated at the 3 h time point should involve genes essential for the action of the first stimulation, because DRB treatment during the initial 3 h inhibited the action of the first stimulation **(** **Figure 7** **)**. Therefore, we identified genes that were specifically regulated at 3 h after the first stimulation by performing a microarray assay. We used transient stimulation with NGF or PACAP and continuous stimulation with NGF as positive controls and transient stimulation with insulin as a negative control, since PACAP and NGF, but not insulin, can serve as transient first stimulants **(** **Figure 6A** **)**. We selected genes that were regulated by these positive stimulants but not by insulin. We also used U0126 and LY294002 to identify genes that were specifically regulated by ERK, but not PI3K, because the action of the first stimulation was blocked by the addition of U0126, but not by that of LY294002 **(** **Figure 5A** **)**. Therefore, we used transient NGF stimulation, with or without either U0126 or LY294002 and selected the genes whose expressions were affected by the addition of U0126, but not by the addition of LY294002.

At the 3-h time point, 223 genes and 37 genes were up- or down-regulated in response to continuous NGF stimulation **(see Table S1);** 176 genes and 28 genes were up- or down-regulated by transient NGF stimulation, while 424 genes and 67 genes were up- or down-regulated by transient PACAP stimulation. We selected the genes that were commonly regulated by the above-described positive stimulations but not by the transient insulin stimulation and identified 82 up-regulated and 8 down-regulated genes. We further narrowed down the potential genes by identifying those whose expressions were affected by the addition of U0126, but not by LY294002. As a result, 47 up-regulated and 3 down-regulated genes were selected **(** **Table 1** **)**.

**Table 1:** Genes identified as responsible for the action of the first stimulation.

Gene Name	RefSeq	Fold change						Reference
		S.NGF	T.NGF	T.PACAP	LY294002	T.Insulin	U0126	
TC598582	TC598582	0.2	0.17	0.17	0.16	1.24	0.31	
CF110295	CF110295	0.1	0.22	0.15	0.05	1.29	0.27	
RGD1560744_predicted	ENSRNOT00000028929	0.25	0.25	0.23	0.2	0.64	0.31	
Emp2	NM_001007721	4.36	4.1	9.69	7.59	1.36	1.77	[30]
A_44_P929787	A_44_P929787	6.11	4.26	5.85	5.07	1.41	3.44	
Dusp5	NM_133578	5.28	4.28	4.45	4.14	1.14	2.21	
LOC302733	XR_005937	6.81	4.35	10.56	5.42	1.33	1.5	
Vip	ENSRNOT00000025477	5.3	4.52	4.7	4.63	1	2.63	
Fscn2_predicted	ENSRNOT00000054975	5.1	4.54	42.2	6.29	1.34	2.02	
Plk2	NM_031821	4.15	4.68	68.3	4.23	1.19	2.06	[30]
AF465254	AF465254	5.54	4.88	6.5	4.63	1.42	3.71	
XM_225257	XM_225257	6.04	5.04	6.96	5.39	1.54	2.61	
Trib1	ENSRNOT00000005885	6.03	5.13	4.88	6.75	1.22	1.94	[30]
DV725280	DV725280	4.14	5.14	55.24	6.17	1.12	2.17	
TC596558	TC596558	7.88	5.83	4.75	4.25	1.42	3.88	
A_44_P316422	A_44_P316422	7.22	5.85	4.41	6.15	1.15	1.93	
Col7a1_predicted	ENSRNOT00000027994	7.71	5.93	7.44	6.39	3.76	2.93	
Prlpf	NM_022530	5.15	6.27	4.19	10.92	1	2.12	
AA875314	AA875314	4.44	6.32	34.71	11.03	1.15	2.15	
CA509943	CA509943	7.55	6.5	4.55	6.5	1.45	1.18	
Igsf9b_predicted	XM_235959	4.52	6.52	9.61	5.84	0.81	1.13	[30]
Metrnl	NM_001014104	5.55	6.6	14.7	7.05	1.1	1.75	
ENSRNOT00000059981	ENSRNOT00000059981	5.45	7.65	19.7	10.35	1.1	2.2	
TC612776	TC612776	9.1	7.75	11.9	18.05	1.35	1.75	
Crem	NM_013086	8.57	8.24	19.48	5.24	1.48	2.71	[30]
Pnoc	NM_013007	6.5	8.39	5.5	26.06	1	2.89	
Ania4	AF030089	7.33	8.61	8.78	26.5	1.17	2.44	[29]
Nucb1	BC062084	12.48	8.91	18.57	4.3	1.13	0.78	
BF562910	BF562910	11.83	9.5	11.06	5.5	1.22	3.89	
ENSRNOT00000042374	ENSRNOT00000042374	7.56	9.92	159.2	13.2	1.08	3.88	
PVR	NM_017076	9.29	10.64	13.5	8.36	2	1.57	[30]
Tph1	ENSRNOT00000056109	8.76	10.86	172.86	7.29	1.62	1.9	
Crisp1	NM_022859	11	11.07	7.07	41.14	1.21	3.79	
Stfa2_predicted	ENSRNOT00000045639	17.52	11.35	4.87	13.13	2.65	0.65	[30]
RGD1306658	NM_001014216	14.37	12.68	40.63	4.84	1.47	0.89	[30]
Ankrd1	NM_013220	13.14	12.93	7	9.79	1.07	2.5	
RGD1310139_predicted	XM_226732	13.71	12.93	11.86	4.21	0.5	1.64	
Serpinb1a	NM_001031642	13.23	14.32	4.32	20.77	1.09	2.45	[31]
Plaur	NM_134352	16.22	15	4.65	15.52	2.09	2.13	[29], [30]
ENSRNOT00000028839	ENSRNOT00000028839	16.81	15.75	5.69	13.06	1.25	3.88	
Homer1	U92079	17.91	17.91	22.82	8.45	1.18	1.82	
BF564195	BF564195	18.7	19.22	4.33	4.56	0.96	2.7	
TC612735	TC612735	14.78	20	58.33	12.44	1.33	2.22	
RGD1305778	NM_001014043	20.85	21.77	24.38	7.62	1.38	1.92	
Dcamkl1	NM_053343	21.25	24.42	52.25	9.42	1.5	1.75	[29], [30]
Rrad	NM_053338	20.63	25.75	17.5	22	1.13	1.5	
AA891911	AA891911	15.25	32.75	86.25	49.38	1.63	1.38	
LOC499660	ENSRNOT00000012174	31.4	35.4	25.6	31.4	1.2	1.6	
Neu2	NM_017130	100	99.23	7.62	273.85	0.85	2.46	
Mmp13	ENSRNOT00000011507	130	179	280	285	1	4	[29]

We selected genes whose expression level showed a greater than 4-fold change over the control in response to each pattern of stimulation, such as transient NGF (T.NGF), PACAP (T.PACAP) and insulin (T.Insulin) stimulation, sustained NGF stimulation (S.NGF) and transient NGF stimulation with U0126 (U0126) or LY294002 (LY294002). Then, we selected genes commonly found to be responsive to transient NGF, transient PACAP, sustained NGF and transient NGF with LY294002, but unresponsive to transient insulin stimulation and transient NGF stimulation with U0126.

The following features suggest the reliability of the gene identification data. First, about 90% of the genes responsive to transient NGF stimulation were also found to be responsive to continuous NGF stimulation. Second, about 80% of the genes responsive to transient NGF and PACAP stimulations were de-regulated by the U0126 treatment, which is consistent with previous reports that NGF and PACAP commonly activate the ERK pathway [18]. Third, 13 of the 50 genes listed in Table 1 have also been identified as NGF-responsive in previous microarray studies, suggesting that the identified genes are highly likely to be involved in the differentiation process of PC12 cells, especially during the action of the first stimulation **(** **Table 1** **)** [29]–[31].

Furthermore, we were able to characterize the identified genes as being related to the extracellular region, endopeptidase inhibitor activity or hormone activity by exploring the gene ontology (GO) classes **(Table 2)**. Altogether, we identified several genes, some of which are responsible for the action of the first stimulation, using a microarray assay followed by the serial gene selection steps described above (See the Discussion for further discussion of the possible mechanism of the latent process.).

**Table 2:** .1 **** Over-represented GO classes of the first-stimulation-responsive genes.

Ontology	GOID	Term	Identified genes^a^	Genes/GOID^b^	*p* value^c^
Biological Process	GO:0042428	serotonin metabolic process	2	6	0.00001
	GO:0046219	indolalkylamine biosynthetic process	2	6	0.00001
	GO:0006568	tryptophan metabolic process	2	7	0.00001
	GO:0016477	cell migration	4	246	0.00161
	GO:0048384	retinoic acid receptor signaling pathway	1	7	0.00284
	GO:0043403	skeletal muscle regeneration	1	7	0.00284
	GO:0042176	regulation of protein catabolic process	2	51	0.00303
	GO:0001764	neuron migration	2	76	0.00714
	GO:0030900	forebrain development	2	79	0.00757
	GO:0030282	bone mineralization	1	17	0.01002
	GO:0035116	embryonic hind limb morphogenesis	1	17	0.01002
	GO:0008544	epidermis development	2	109	0.01481
	GO:0000270	peptidoglycan metabolic process	1	23	0.01554
	GO:0051017	actin filament bundle formation	1	23	0.01554
	GO:0042311	Vasodilation	1	24	0.01649
	GO:0000902	cell morphogenesis	3	298	0.01934
	GO:0009888	tissue development	4	543	0.02028
	GO:0030216	keratinocyte differentiation	1	30	0.02341
	GO:0030162	regulation of proteolysis	1	31	0.02471
	GO:0009612	response to mechanical stimulus	1	37	0.03151
	GO:0051216	cartilage development	1	45	0.04294
	GO:0009653	anatomical structure morphogenesis	5	1035	0.04612
Cellular Component	GO:0030934	anchoring collagen	1	12	0.00644
	GO:0005576	extracellular region	9	2268	0.01554
	GO:0046658	anchored to plasma membrane	1	23	0.01554
	GO:0005793	ER-Golgi intermediate compartment	1	28	0.02087
	GO:0043005	neuron projection	3	331	0.02641
	GO:0005667	transcription factor complex	2	192	0.04855
Molecular Function	GO:0004866	endopeptidase inhibitor activity	4	166	0.00031
	GO:0001515	opioid peptide activity	1	5	0.00165
	GO:0004308	exo-alpha-sialidase activity	1	5	0.00165
	GO:0004869	cysteine-type endopeptidase inhibitor activity	2	41	0.00186
	GO:0005179	hormone activity	3	152	0.00313
	GO:0016597	amino acid binding	2	58	0.00408
	GO:0008147	structural constituent of bone	1	9	0.00413
	GO:0017017	MAP kinase tyrosine/serine/threonine phosphatase activity	1	10	0.0048
	GO:0005452	inorganic anion exchanger activity	1	12	0.00644
	GO:0004867	serine-type endopeptidase inhibitor activity	2	95	0.01098
	GO:0004497	monooxygenase activity	2	122	0.01848
	GO:0005102	receptor binding	5	895	0.02685
	GO:0005184	neuropeptide hormone activity	1	34	0.0284
	GO:0005057	receptor signaling protein activity	2	170	0.03886
	GO:0016301	kinase activity	5	990	0.04
	GO:0005515	protein binding	16	6578	0.04321

A GO enrichment analysis was performed using the web-based GOEAST software toolkit with a significance threshold of *p* = 0.05. ^c^ *p* values less than 0.05 indicate GO classes that are more than 95% likely to be overrepresented. ^a^Number of genes in the indicated GOID class regulated by the first stimulation. ^b^Total number of genes in the indicated GOID class present on the microarray.

1 Abbreviations: GO, Gene Ontology; GOID, Gene Ontology Identification.

## Discussion

**Figure 8:**
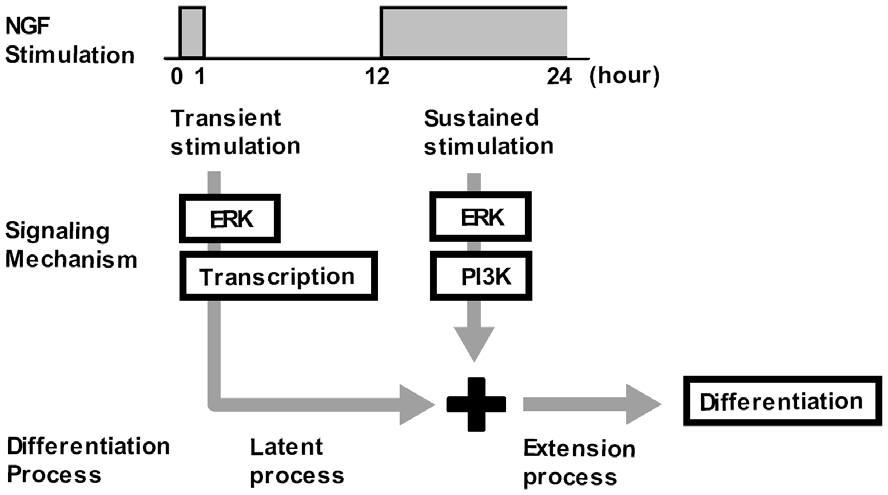
Summary diagram of the actions of NGF. The first transient NGF stimulation drives the latent process via ERK and transcription activities, while the second sustained NGF stimulation drives the fast extension process via ERK and PI3K activities. Thus, NGF induces cell differentiation via two temporally and mechanistically distinct processes.

To disclose the mechanism underlying the continuous stimulation requirement for PC12 cell differentiation, we performed a discontinuous stimulation assay. We found that discontinuous stimulations were sufficient to induce normal cell differentiation **(** **Figure 8** **)**. The first transient stimulation induced an ERK- and transcription-dependent latent process, while the second sustained stimulation induced an ERK- and PI3K-dependent fast extension process. We propose that continuous NGF stimulation is required to induce two temporally separated differentiation processes: the latent and extension processes.

The need for discontinuous stimulations has been found in other biological processes [12], [32]–[34]. Discontinuous stimulation is sufficient for serum-arrested fibroblasts to enter S phase [12]. The commitment of naïve T cells to proliferation is also induced by discontinuous stimulations [33]. Our results, together with the results of these previous studies, show that a need for discontinuous growth factor stimulations can be a general mechanism driving biological processes, such as differentiation and proliferation.

A discontinuous stimulation assay is valuable for investigating temporally distinct processes involved in a certain biological event. Indeed, we were able to investigate and understand the underlying signaling mechanisms (i.e., the temporal requirement of a particular signaling activity) of the latent and extension processes.

The latent process is mediated by ERK and transcriptional activity upon transient NGF stimulation and requires about 12 h for optimal effect. A distinctive feature of the latent process is that transient stimulation is sufficient for its induction. One and one-half min of NGF stimulation was sufficient to induce the latent process. In contrast, EGF could not replace NGF as the first stimulant, even though an important difference in the EGF- and NGF-signaling cascades is their sustainability: EGF transiently stimulates a downstream signaling cascade, whereas NGF induces the sustained activation of a signaling cascade [8], [19], [35]. The characteristics of TrkA trafficking might explain this contradiction [36]. The activity of TrkA after transient NGF stimulation can continue for a couple of hours because of its slow degradation kinetics, resulting in the sustained activation of downstream molecules for about two hours **(see Figure S8).** In light of the findings of Jullienj *et al.*, which demonstrated the importance of sustained ERK activity for differentiation [28], [37], sustained ERK activity for 2 h appeared to be important for the induction of the latent process. The fact that PACAP, but not bFGF, EGF, insulin or forskolin, was capable of inducing the latent process also supports this idea, since a common feature of NGF- and PACAP-dependent signaling cascades, but not of those of other growth factors, is the sustained activation of ERK and CREB. Taken together, the studies' findings indicate that transient NGF stimulation is sufficient for the sustained activation of downstream molecules such as ERK, which results in the induction of a 12 h long latent process. Such long latent process can be triggered by the transient addition of NGF.

About 12 h after the first stimulation, a second sustained stimulation induced a fast neurite extension process that is ERK- and PI3K-dependent, but not transcription-dependent, indicating a synergistic function of the latent process and the second stimulation. One feature of the second stimulation is its temporal requirement. The existence of an interval with an optimal duration suggests that the neurite extension rate is affected by the progression of the latent process, such as the accumulation of effector molecules. The idea that the accumulation of effector molecules was induced by the first stimulation and that the first stimulation provides the prerequisite conditions of fast neurite extension by the second stimulation may account for the faster neurite extension in response to discontinuous, rather than continuous, stimulation. Another feature of the induction of the extension process is its requirement for sustained stimulation, suggesting the need for sustained signaling activities, such as ERK, PI3K or other molecules. The need for a sustained regulation of a post-transcriptional mechanism, such as cytoskeletal regulation, for continuous neurite extension could be an underlying reason. The need for sustained NGF stimulation and PI3K activities [38]–[40] during cell differentiation is probably associated with the extension process, rather than the latent process.

The different signaling mechanisms for the latent and extension processes have the following remarkable features. First, although the activity of ERK is required during both processes, the likely roles of ERK appear to differ for each process. ERK regulates the latent process in a transcription-dependent manner, as indicated by the microarray assay **(see Table S1),** whereas ERK regulates the extension process in a transcription-independent manner **(** **Figure 5A** **)**, indicating context-dependent**,** diverse functions of ERK for cell differentiation. Second, the activity of PI3K is required only during the extension process, suggesting that the sustained activity, rather than the initial activity, of PI3K on continuous NGF stimulation is important for cell differentiation. The mechanism of PI3K activity in PC12 cell differentiation is a novel finding. We cannot address this kind of question with a continuous stimulation assay. The need for PI3K activity for the extension process is consistent with previous studies that have demonstrated the necessity of PI3K for the morphological differentiation of PC12 cells [38]–[39], [41]–[42]. Third, transcriptional activity is required only during the latent process. This finding is consistent with that of previous studies reporting the importance of transcriptional activity during the initial response to NGF [28]. However, it is noteworthy that the extension process occurs in a manner that is independent of transcription. This result suggests that the second stimulation regulates post-transcriptional mechanisms (e.g., translation of pre-existing genes, protein phosphorylation, or the regulation of protein-protein interactions) resulting in the induction of neurite extension. Note that this result does not mean that transcriptional activity is unnecessary for subsequent differentiation processes, given that we explored, in this study, a differentiation process that occurs within 24 h of the initial NGF stimulation.

To gain deeper insight into the induction mechanism of the latent process, we identified genes potentially involved in this process. Microarray assays are prone to the identification of false-positive genes. To address this problem, we extracted RNA at particular time points and used diverse stimulation patterns. This method allowed us to identify genes that were highly likely to be involved in the latent process with a high reliability.

We performed a GO analysis to further understand the detailed biological events involved in the latent process. The 50 identified genes were categorized in several GO classes, such as extracellular region, endopeptidase inhibitor, tissue development and cell migration **(Table 2)**. Plaur, Mmp13 and Col7a1 are included in both the extracellular region and tissue development GO classes. These gene products are commonly associated with extracellular membrane (ECM) formation, which is important for cell differentiation. Plaur encodes urokinase receptor (uPAR), which converts plasminogen into plasmin, encodes serine proteinase, and regulates ECM formation and signaling activation, resulting in the regulation of diverse biological processes including PC12 cell differentiation [16]–[17], [43]. Matrix metalloproteinase13 (MMP13) regulates ECM formation, signaling activity and other processes [44]–[45]. Col7a1, a type VII collagen, is the main component of anchoring fibrils and requires proteolytic activation for its conversion into mature anchoring fibril collagen [46]–[47]. Similarly, Plaur and PVR are typically included in the extracellular region and cell migration classes. PVR encodes transmembrane glycoprotein, which mediates cell attachment to the ECM [48]. Furthermore, Plaur and Serpinb1a are typically included in the GO class for the regulation of protein catabolic processes. Serpinb1a exhibits a serine-type endopeptidase inhibitor activity, which might negatively regulates the action of uPAR [49]. Taken together, the findings of the GO analysis suggest that appropriate ECM formation by these gene products or the activation of latent growth factors followed by signaling activation could play critical roles during the latent process. We found VIP in the GO classes for extracellular region and hormone activity. VIP, which is a peptide similar to PACAP, has been reported as a differentiation factor of PC12 cells and other neuronal cells [50]–[51]. Therefore, the action of the first stimulation could be mediated by the autocrine or paracrine activity of VIP. These findings indicate that an autocrine or paracrine loop of extracellular proteins is one possible molecular mechanism responsible for the latent process.

Our findings demonstrate that the differentiation process of PC12 cells consists of temporally separated latent and extension processes. Distinct temporal patterns of NGF stimulation (timing and duration of the stimulation) are necessary for the induction of each process and for cell differentiation. Discontinuous NGF stimulation is sufficient to induce PC12 cell differentiation.

## Supporting Information

Figure S1 Full differentiation of PC12 cells after discontinuous stimulation. PC12 cells were transiently (1 h), continuously or discontinuously (1 h of first stimulation and a sustained second stimulation beginning at the 12 h time point) stimulated with 50 ng/mL of NGF. (A) Phase contrast images were taken 8 days after stimulation. (B) mRNA expression levels of NF-L, TGFβ, VGF, PVR, Plk2 and Plaur on the indicated days after the initial stimulation were measured using real-time PCR. The data were normalized using the expression level of ACTB as an internal control. The mRNA expression levels of non-stimulated cells were used as a control. The values represent the mean fold expression compared with the control ± S.E.M. (n = 3).
(3.75 MB TIF)

Figure S2 Neurite extension after optimal and suboptimal conditions of discontinuous stimulation. (A-I) Distribution patterns of neurite lengths. Histograms of the neurite lengths of individual cells (total neurite lengths) (A) before stimulation, (B,D) 12 h after (B) continuous or (D) discontinuous stimulation, and (C,E-G) 24 h after (C) continuous or discontinuous stimulation with (E) the default condition, (F) 0.5 ng/mL of NGF for the first stimulation, or (G) the second stimulation treated for 1 h are shown. (H) The frequency of panel (E) was subtracted from that of panel (F), indicating the relative frequency. (I) The frequency of panel (E) was subtracted from that of panel (G), indicating the relative frequency. (J) Neurite lengths after continuous or discontinuous NGF stimulation. PC12 cells were exposed to NGF-free medium (open circle) or continuous stimulation with 50 ng/mL of NGF (closed circle), discontinuous stimulation for 12 h (closed square) or 1 h for the second 50-ng/mL NGF stimulation (open square). At the indicated time points, the neurite lengths were measured as described in Materials and Methods. The values represent the mean ± S.E.M. (n = 3).
(5.41 MB TIF)

Figure S3 Effects of various inhibitors on continuous stimulation-dependent neurite extension. PC12 cells were pretreated with or without the indicated inhibitors for 20 minutes, then treated with 50 ng/mL of NGF for 24 hours in the presence of each inhibitor. The cells were fixed with formalin, and the mean neurite lengths were measured as described in Materials and Methods. The values represent the mean ± S.E.M. (n  = 3). *p<0.05; Student t-test comparing DMSO- and inhibitor-treated cells.
(1.23 MB TIF)

Figure S4 Reversible effects of inhibitors on signaling activation. To confirm the reversibility of the inhibitor effects, sequential treatment was performed after pre-treatment with the indicated inhibitors: (A, B) k252a (200 nM); (C) U0126 (50 nM); (D) LY294002 (50 nM). The cells were then washed and stimulated with 50 ng/mL of NGF. For inhibitor pretreatment, the cells were incubated with the indicated inhibitors for 20 minutes. Washing out was performed as described in Materials and Methods. A total of 50 ng/mL of NGF was added with or without an inhibitor for 5 minutes. After the sequential performance of the indicated treatment combinations, the activities of the indicated signaling molecules were measured as described in Materials and Methods.
(4.29 MB TIF)

Figure S5 Neurite extension upon continuous stimulation with various stimulants. PC12 cells were continuously stimulated with NGF (50 ng/mL), PACAP (100 nM), Forskolin (10 µM), insulin (10 nM), EGF (50 ng/mL) or bFGF (50 ng/mL) for 24 hours. Then, the cells were fixed with formalin, and the mean neurite lengths were measured as described in Materials and Methods. Values represent the mean ± S.E.M. (n = 3) of the neurite extension length during 24 hours.
(1.08 MB TIF)

Figure S6 Activities of ERK, CREB and Akt after treatment with various stimulants. Time course intensity of the phosphorylation of (A) pERK, (B) pCREB, and (C) pAkt were quantified as described in Materials and Methods after continuous stimulation of the PC12 cells with the indicated stimulants. (D-I) Immunocytochemical images used to quantify the molecular activities after stimulation with (D) 50 ng/mL of NGF, (E) 100 nM PACAP, (F) 10 nM insulin, (G) 50 ng/mL EGF, (H) 50 ng/mL bFGF, or (I) 10 uM of forskolin.
(1.60 MB PDF)

Figure S7 Images used to quantify neurite lengths. Using the CellMask signal as a neuronal image and the Hoechst signal as a nuclear image, we measured the length of the neurites using the NeuroTracer, Image J plug-in. The average neurite length was calculated by dividing total length of the traced lines by the total number of cells.
(0.34 MB TIF)

Figure S8 Activity of pTrkA after transient NGF stimulation. PC12 cells were stimulated with 50 ng/mL of NGF for 5 min and washed out as described in Material and Methods. The time course data for the pTrkA and ERK signals were then measured as described in Materials and Methods at the indicated time points.
(0.16 MB TIF)

Table S1 Numbers of identified genes. The numbers of genes regulated by diverse patterns of stimulations (>4-fold change) are indicated in the table. “+” denotes the number of genes that were regulated by a more than 4-fold change in response to the indicated stimuli, whereas “−” denotes the number of genes that were regulated by less than a 4-fold change by the indicated stimuli. Genes commonly regulated were counted when more than two stimulants were indicated.
(0.05 MB DOC)
