# Supplementary figures and images for: Timing-Dependent Actions of NGF Required for Cell Differentiation
Source: PLoS One. 2010 Feb 2;5(2):e9011. doi: 10.1371/journal.pone.0009011 (PMC2814856; doi:10.1371/journal.pone.0009011)

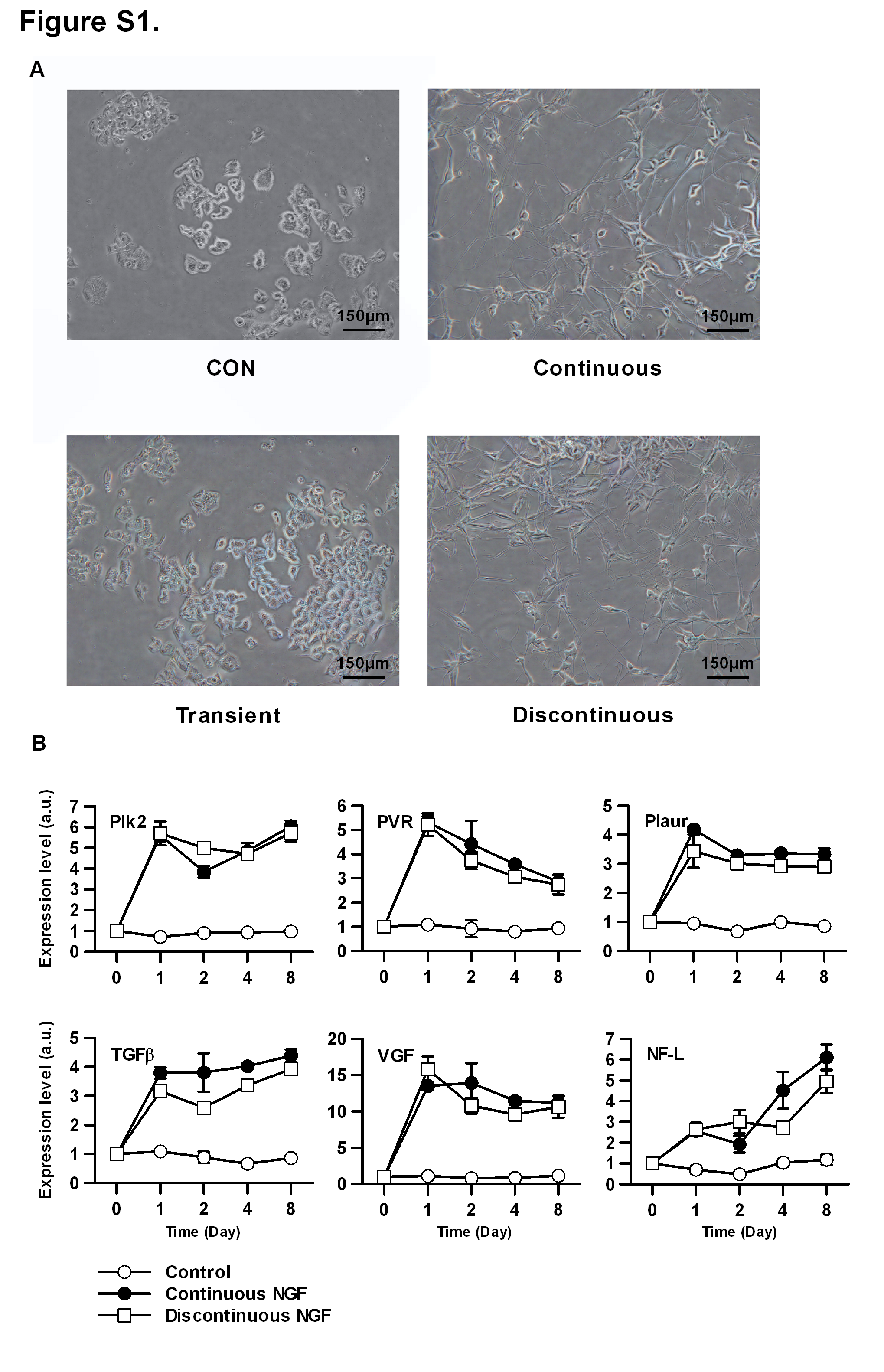

Supplement: Figure S1 — Full differentiation of PC12 cells after discontinuous stimulation. PC12 cells were transiently (1 h), continuously or discontinuously (1 h of first stimulation and a sustained second stimulation beginning at the 12 h time point) stimulated with 50 ng/mL of NGF. (A) Phase contrast images were taken 8 days after stimulation. (B) mRNA expression levels of NF-L, TGFβ, VGF, PVR, Plk2 and Plaur on the indicated days after the initial stimulation were measured using real-time PCR. The data were normalized using the expression level of ACTB as an internal control. The mRNA expression levels of non-stimulated cells were used as a control. The values represent the mean fold expression compared with the control ± S.E.M. (n = 3). (3.75 MB TIF) [file pone.0009011.s001.tif]

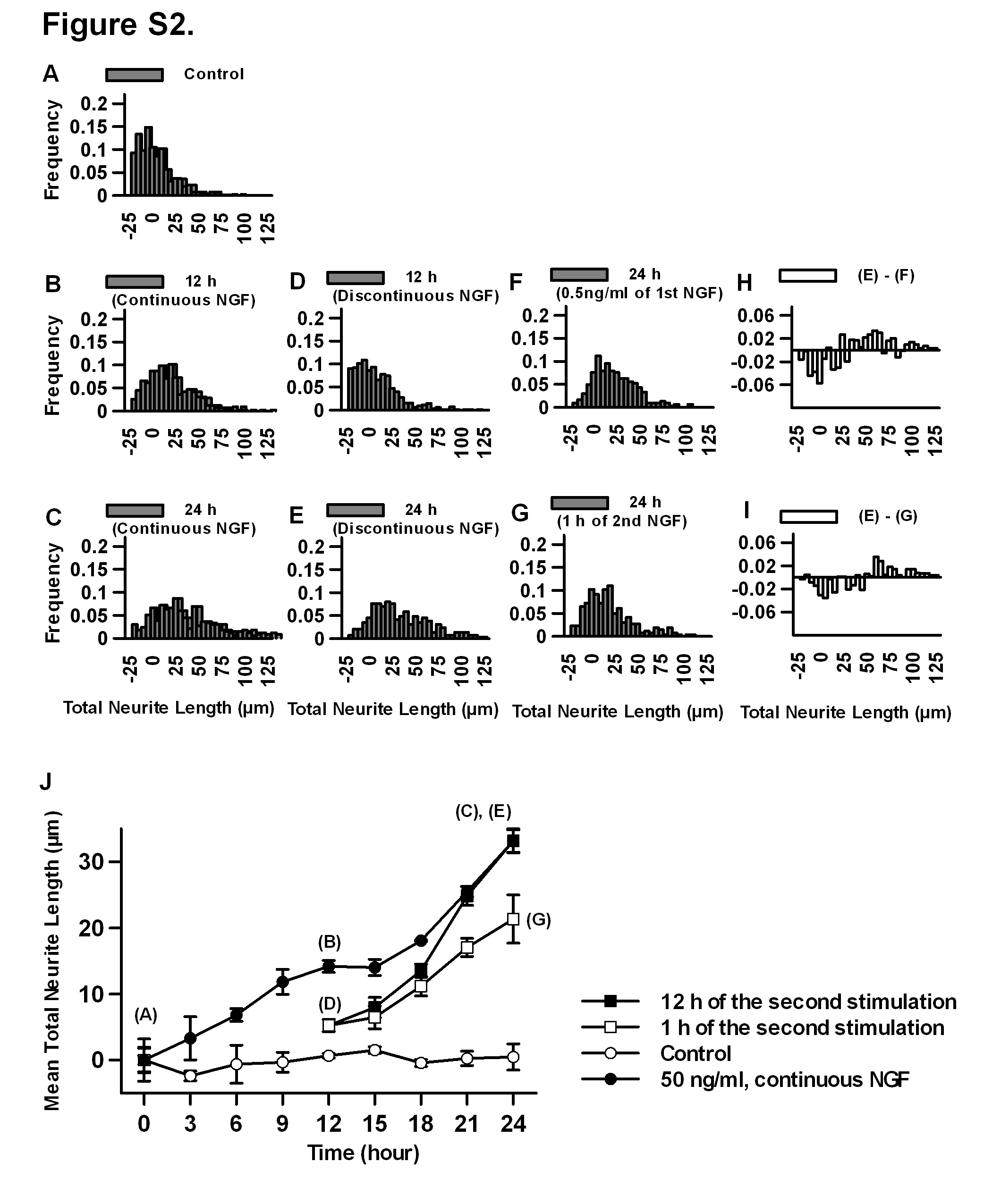

Supplement: Figure S2 — Neurite extension after optimal and suboptimal conditions of discontinuous stimulation. (A-I) Distribution patterns of neurite lengths. Histograms of the neurite lengths of individual cells (total neurite lengths) (A) before stimulation, (B,D) 12 h after (B) continuous or (D) discontinuous stimulation, and (C,E-G) 24 h after (C) continuous or discontinuous stimulation with (E) the default condition, (F) 0.5 ng/mL of NGF for the first stimulation, or (G) the second stimulation treated for 1 h are shown. (H) The frequency of panel (E) was subtracted from that of panel (F), indicating the relative frequency. (I) The frequency of panel (E) was subtracted from that of panel (G), indicating the relative frequency. (J) Neurite lengths after continuous or discontinuous NGF stimulation. PC12 cells were exposed to NGF-free medium (open circle) or continuous stimulation with 50 ng/mL of NGF (closed circle), discontinuous stimulation for 12 h (closed square) or 1 h for the second 50-ng/mL NGF stimulation (open square). At the indicated time points, the neurite lengths were measured as described in Materials and Methods. The values represent the mean ± S.E.M. (n = 3). (5.41 MB TIF) [file pone.0009011.s002.tif]

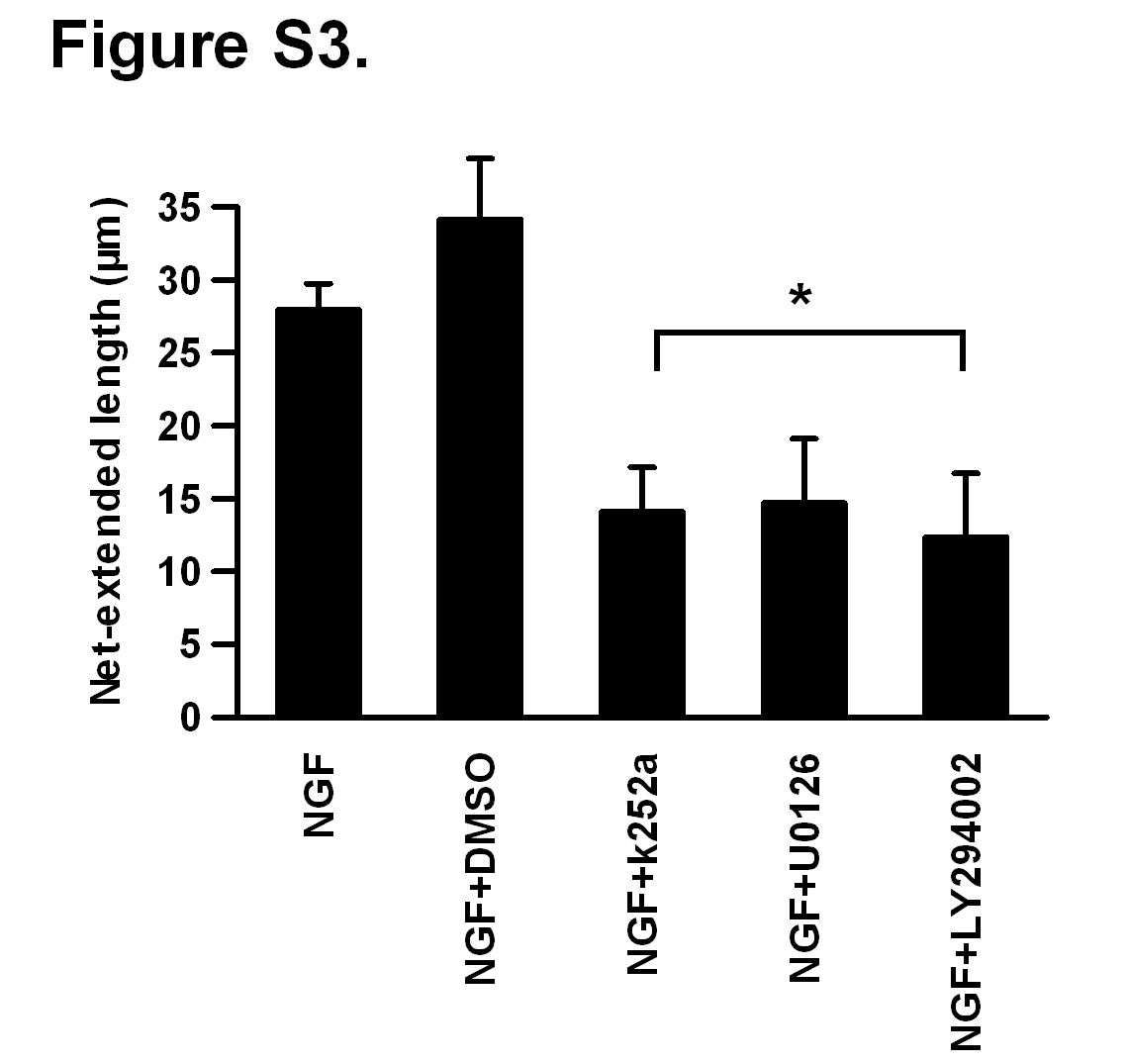

Supplement: Figure S3 — Effects of various inhibitors on continuous stimulation-dependent neurite extension. PC12 cells were pretreated with or without the indicated inhibitors for 20 minutes, then treated with 50 ng/mL of NGF for 24 hours in the presence of each inhibitor. The cells were fixed with formalin, and the mean neurite lengths were measured as described in Materials and Methods. The values represent the mean ± S.E.M. (n = 3). *p<0.05; Student t-test comparing DMSO- and inhibitor-treated cells. (1.23 MB TIF) [file pone.0009011.s003.tif]

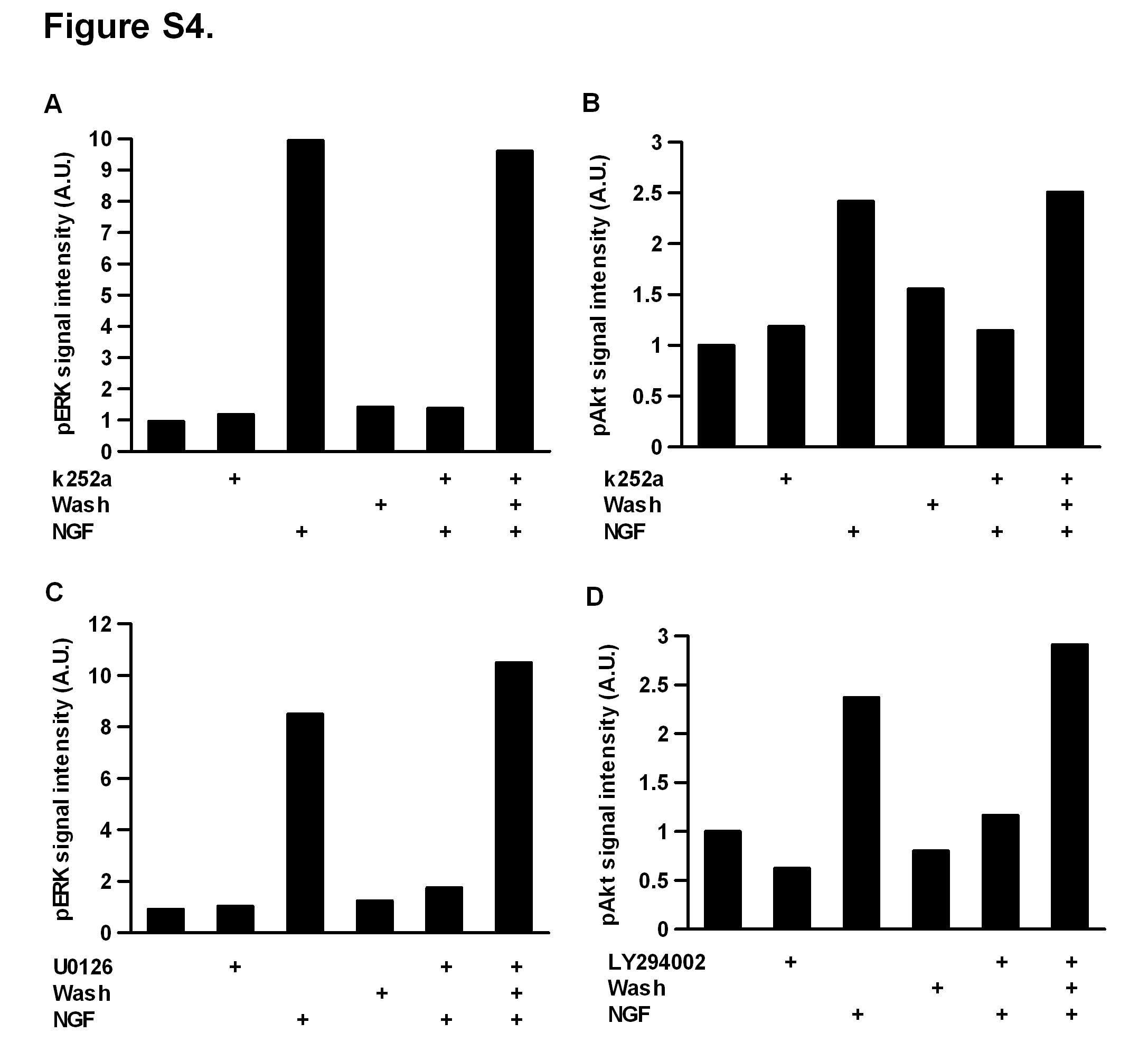

Supplement: Figure S4 — Reversible effects of inhibitors on signaling activation. To confirm the reversibility of the inhibitor effects, sequential treatment was performed after pre-treatment with the indicated inhibitors: (A, B) k252a (200 nM); (C) U0126 (50 nM); (D) LY294002 (50 nM). The cells were then washed and stimulated with 50 ng/mL of NGF. For inhibitor pretreatment, the cells were incubated with the indicated inhibitors for 20 minutes. Washing out was performed as described in Materials and Methods. A total of 50 ng/mL of NGF was added with or without an inhibitor for 5 minutes. After the sequential performance of the indicated treatment combinations, the activities of the indicated signaling molecules were measured as described in Materials and Methods. (4.29 MB TIF) [file pone.0009011.s004.tif]

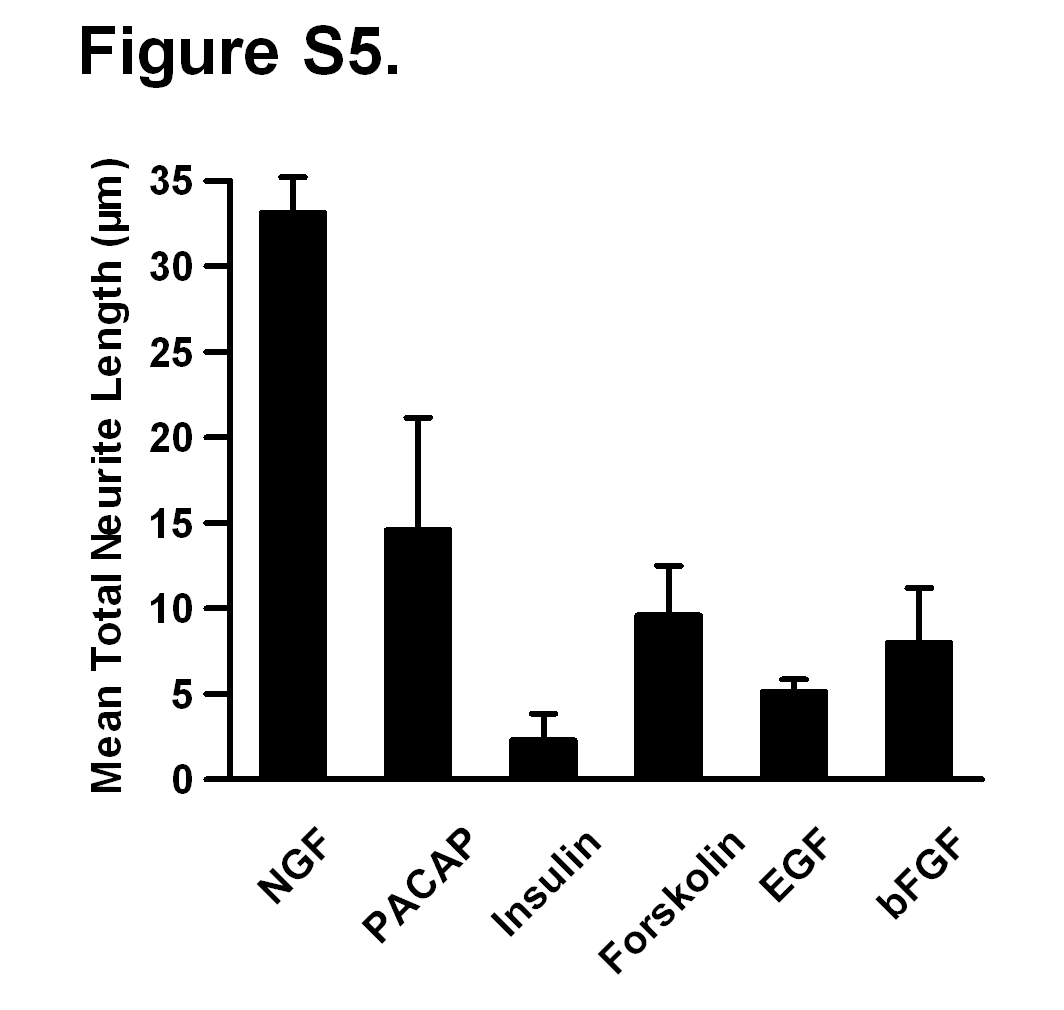

Supplement: Figure S5 — Neurite extension upon continuous stimulation with various stimulants. PC12 cells were continuously stimulated with NGF (50 ng/mL), PACAP (100 nM), Forskolin (10 µM), insulin (10 nM), EGF (50 ng/mL) or bFGF (50 ng/mL) for 24 hours. Then, the cells were fixed with formalin, and the mean neurite lengths were measured as described in Materials and Methods. Values represent the mean ± S.E.M. (n = 3) of the neurite extension length during 24 hours. (1.08 MB TIF) [file pone.0009011.s005.tif]

Figure S6.

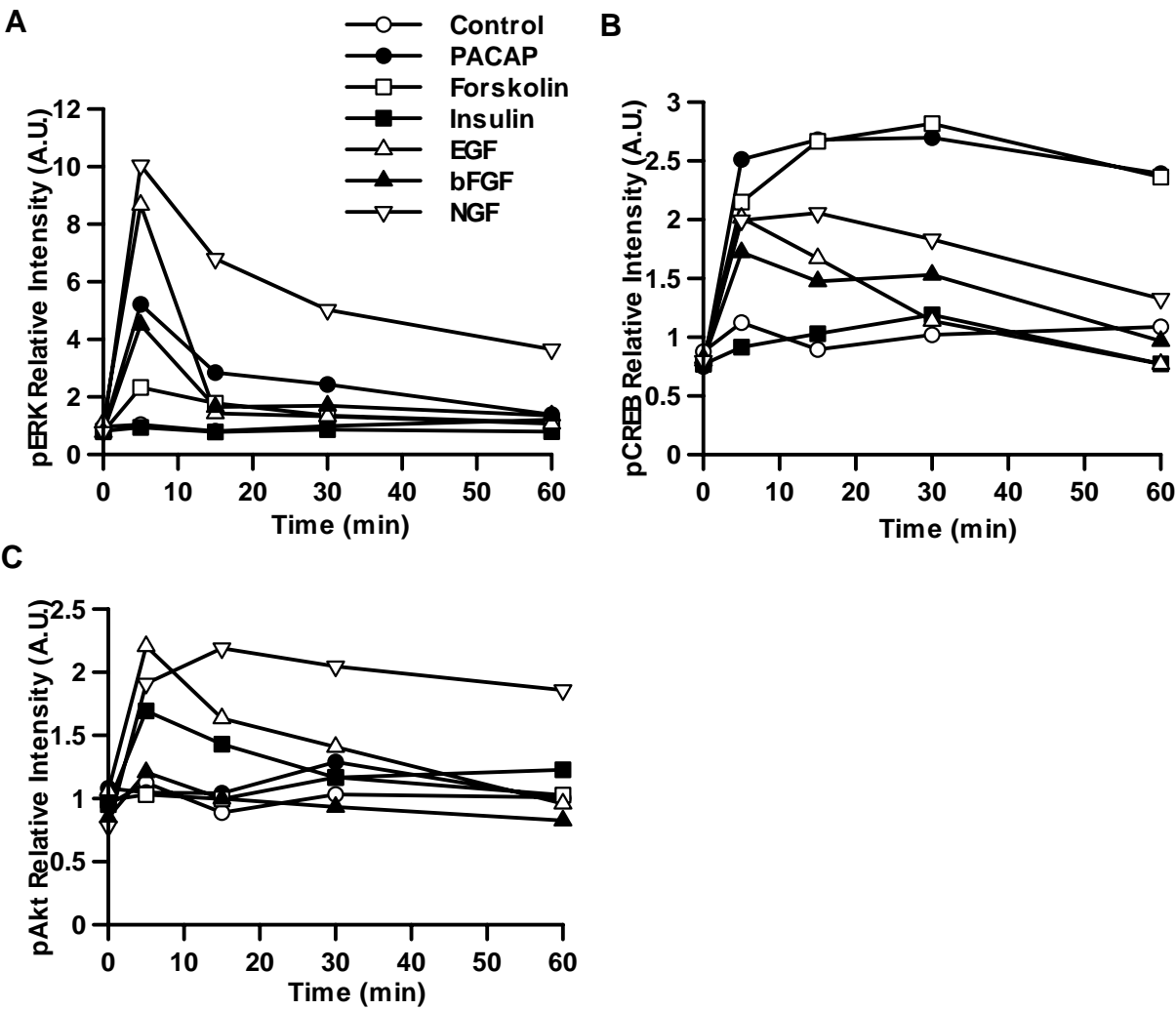

D

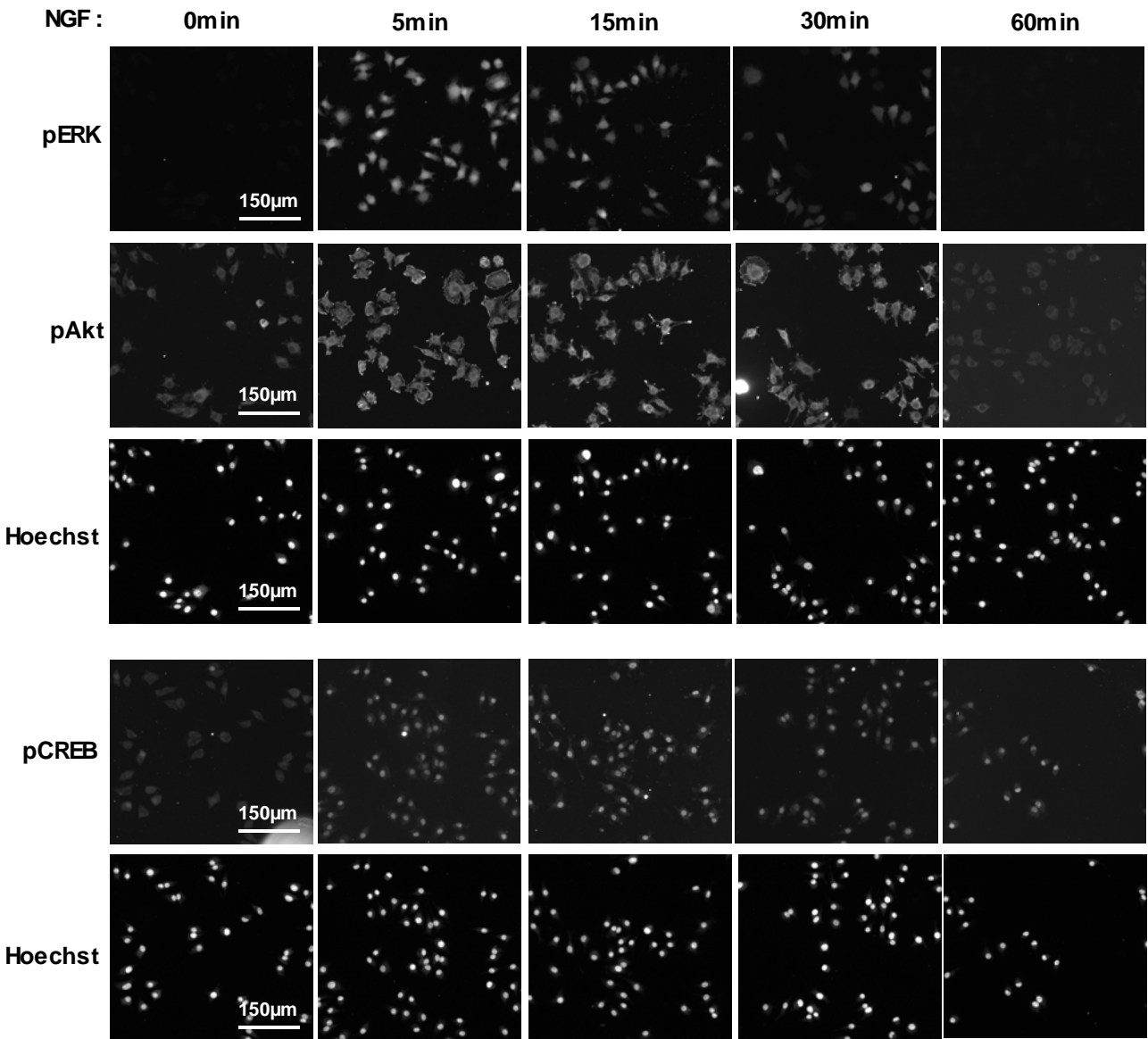

E

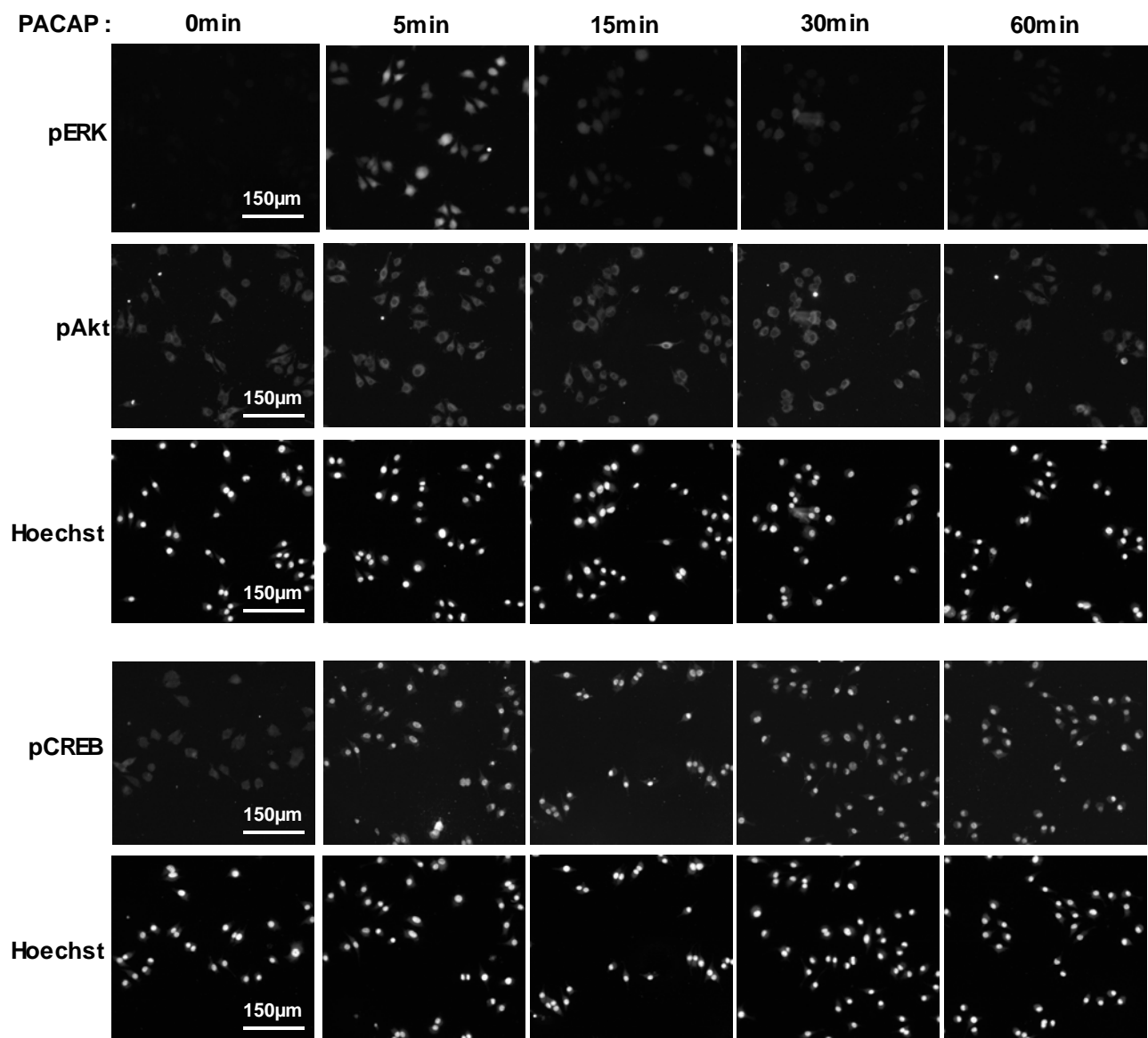

F

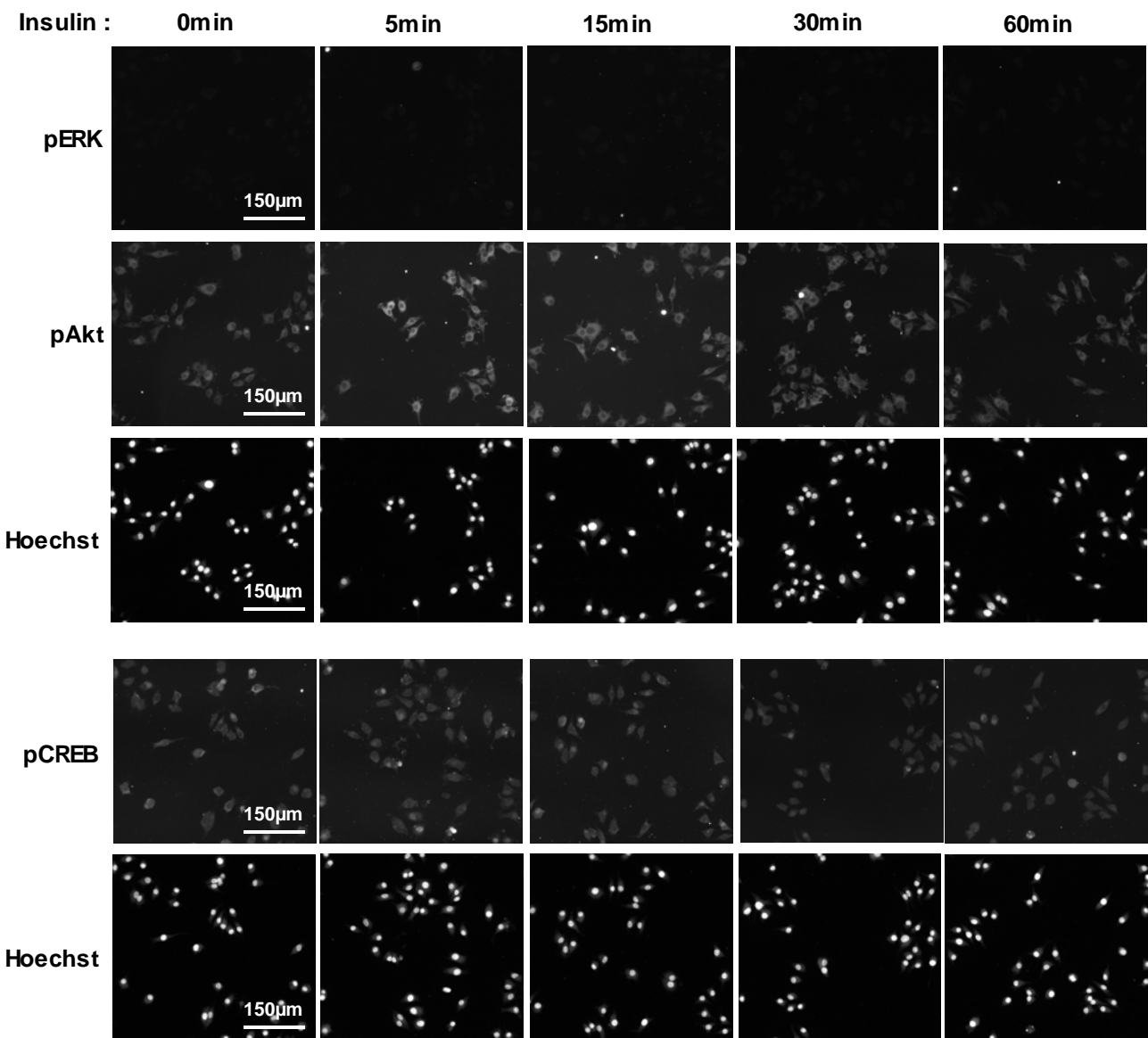

G

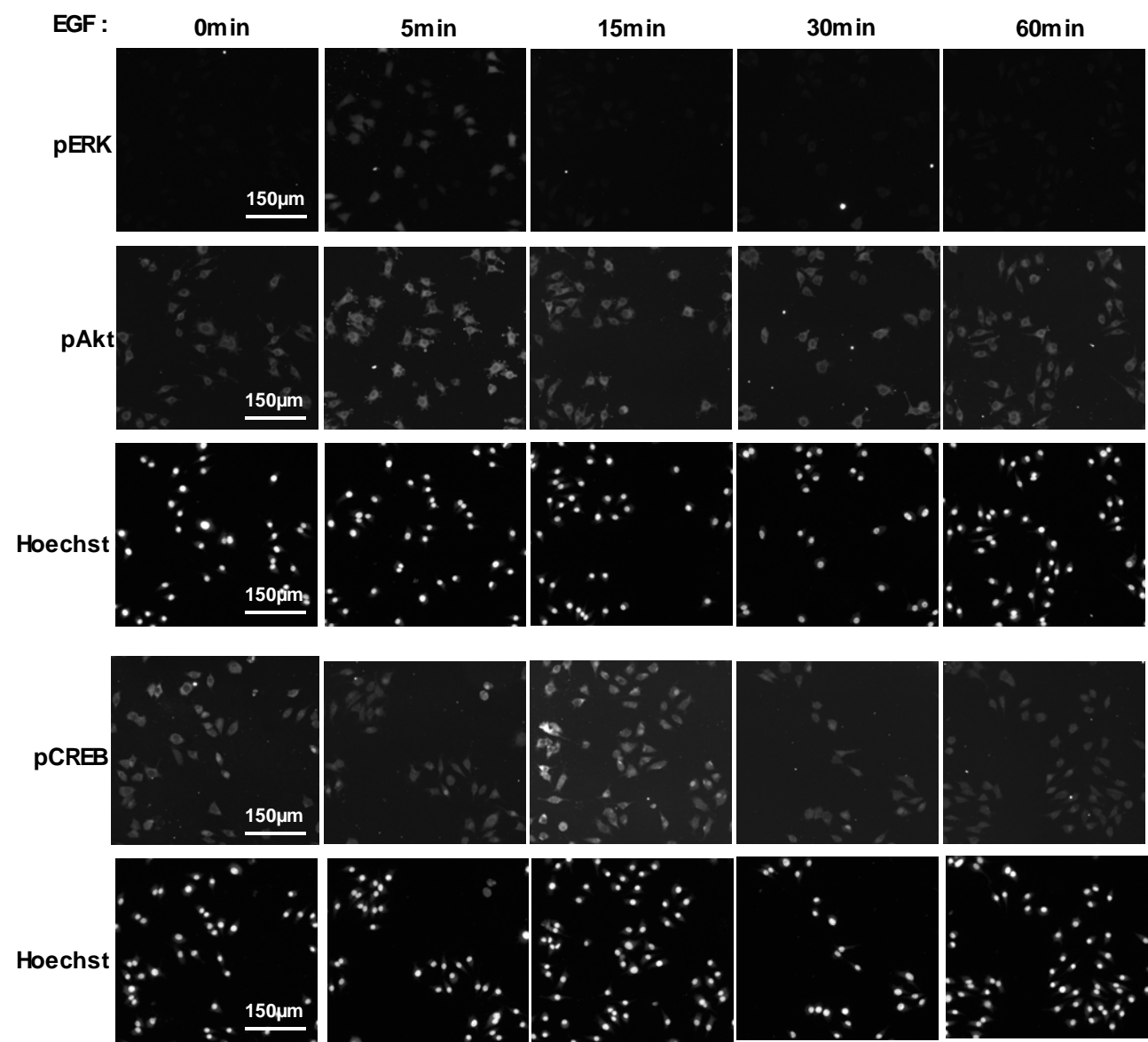

H

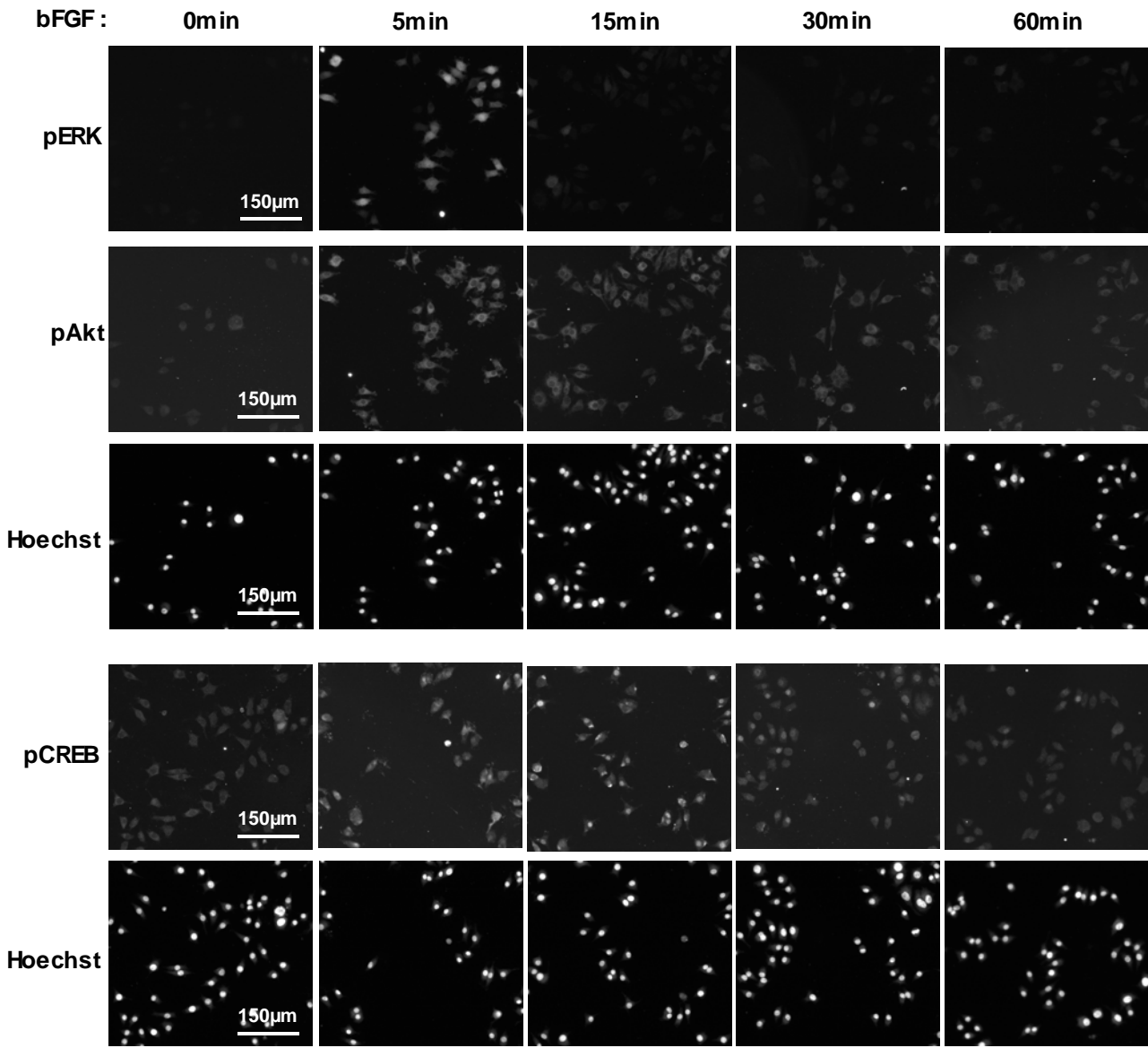

I

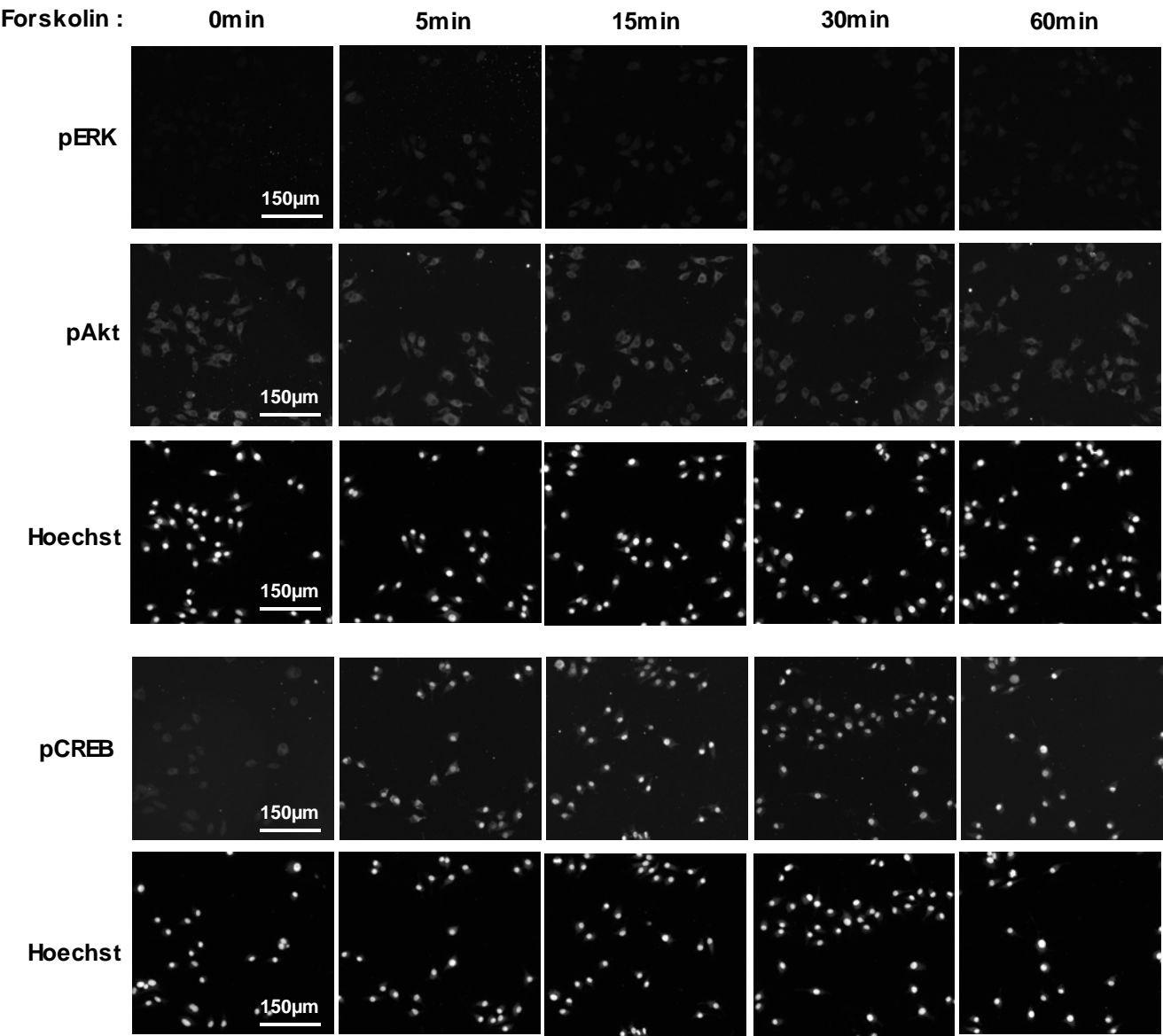

Supplement: Figure S6 — Activities of ERK, CREB and Akt after treatment with various stimulants. Time course intensity of the phosphorylation of (A) pERK, (B) pCREB, and (C) pAkt were quantified as described in Materials and Methods after continuous stimulation of the PC12 cells with the indicated stimulants. (D-I) Immunocytochemical images used to quantify the molecular activities after stimulation with (D) 50 ng/mL of NGF, (E) 100 nM PACAP, (F) 10 nM insulin, (G) 50 ng/mL EGF, (H) 50 ng/mL bFGF, or (I) 10 uM of forskolin. (1.60 MB PDF) [file pone.0009011.s006.pdf]

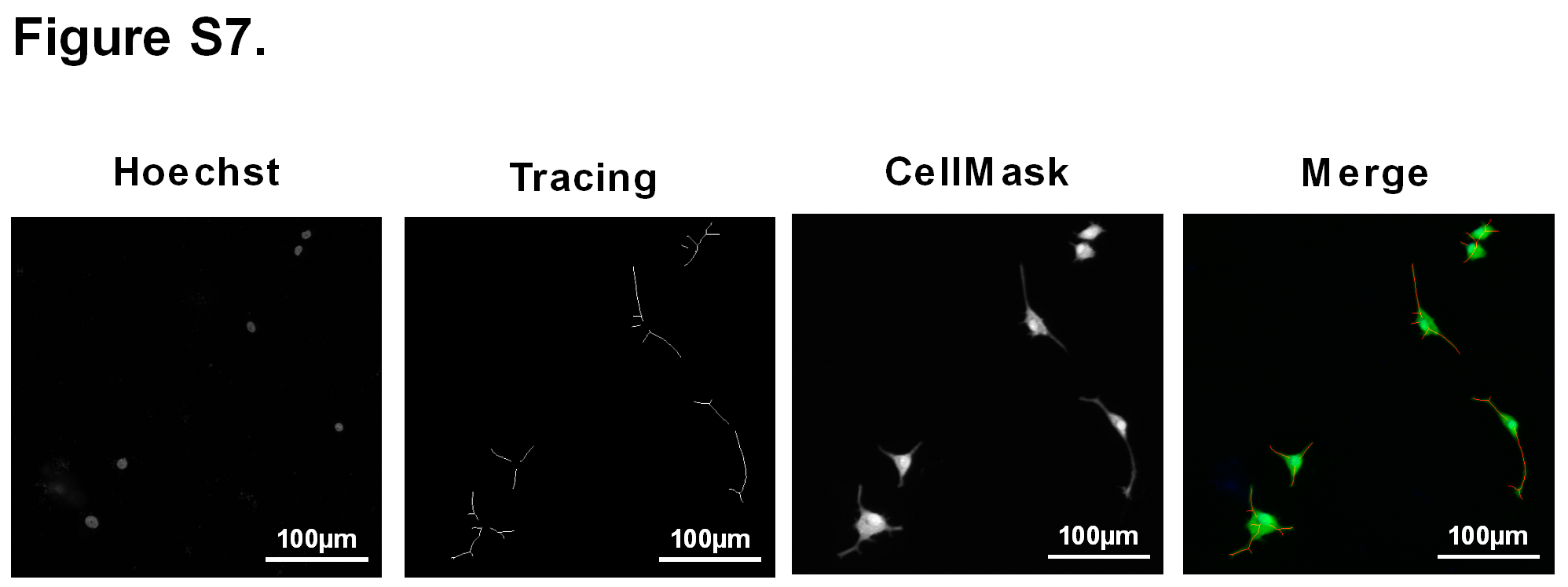

Supplement: Figure S7 — Images used to quantify neurite lengths. Using the CellMask signal as a neuronal image and the Hoechst signal as a nuclear image, we measured the length of the neurites using the NeuroTracer, Image J plug-in. The average neurite length was calculated by dividing total length of the traced lines by the total number of cells. (0.34 MB TIF) [file pone.0009011.s007.tif]

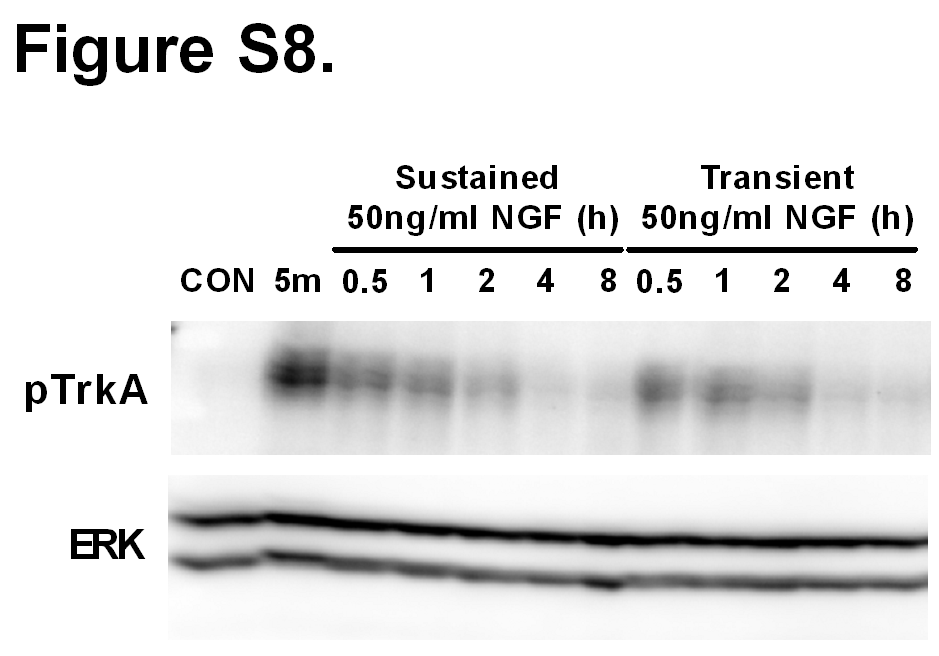

Supplement: Figure S8 — Activity of pTrkA after transient NGF stimulation. PC12 cells were stimulated with 50 ng/mL of NGF for 5 min and washed out as described in Material and Methods. The time course data for the pTrkA and ERK signals were then measured as described in Materials and Methods at the indicated time points. (0.16 MB TIF) [file pone.0009011.s008.tif]
