# Supplementary material for: Timing-Dependent Actions of NGF Required for Cell Differentiation
Source: PLoS One. 2010 Feb 2;5(2):e9011. doi: 10.1371/journal.pone.0009011 (PMC2814856; doi:10.1371/journal.pone.0009011)
Supplement: Table S1 — Numbers of identified genes. The numbers of genes regulated by diverse patterns of stimulations (>4-fold change) are indicated in the table. “+” denotes the number of genes that were regulated by a more than 4-fold change in response to the indicated stimuli, whereas “−” denotes the number of genes that were regulated by less than a 4-fold change by the indicated stimuli. Genes commonly regulated were counted when more than two stimulants were indicated. (0.05 MB DOC) [file pone.0009011.s009.doc]

**Table S1.**

| Stimulation set | | | | | | Number of  identified genes | | |
| --- | --- | --- | --- | --- | --- | --- | --- | --- |
| Continuous  NGF | Transient  NGF | Transient  PACAP | Transient  Insulin | LY294001 | U0126 | Total | Up | Down |
| + |  |  |  |  |  | 260 | 223 | 37 |
|  | + |  |  |  |  | 204 | 176 | 28 |
|  |  | + |  |  |  | 491 | 424 | 67 |
|  |  |  | + |  |  | 77 | 62 | 15 |
|  | + |  |  | + |  | 111 | 95 | 16 |
|  | + |  |  |  | - | 140 | 124 | 16 |
| + | + | + | - |  |  | 90 | 82 | 8 |
| + | + | + | - | + | - | 50 | 47 | 3 |
